# Oncogenic Cell Tagging and Single-Cell Transcriptomics Reveal Cell Type–Specific and Time-Resolved Responses to *Vhl* Inactivation in the Kidney

**DOI:** 10.1158/0008-5472.CAN-23-3248

**Published:** 2024-03-19

**Authors:** Samvid Kurlekar, Joanna D.C.C. Lima, Ran Li, Olivia Lombardi, Norma Masson, Ayslan B. Barros, Virginia Pontecorvi, David R. Mole, Christopher W. Pugh, Julie Adam, Peter J. Ratcliffe

**Affiliations:** 1 Nuffield Department of Medicine, University of Oxford, Oxford, United Kingdom.; 2 Ludwig Institute for Cancer Research, Nuffield Department of Medicine, University of Oxford, Oxford, United Kingdom.; 3 The Francis Crick Institute, 1 Midland Road, London, United Kingdom.

## Abstract

Defining the initial events in oncogenesis and the cellular responses they entrain, even in advance of morphologic abnormality, is a fundamental challenge in understanding cancer initiation. As a paradigm to address this, we longitudinally studied the changes induced by loss of the tumor suppressor gene von Hippel Lindau (*VHL*), which ultimately drives clear cell renal cell carcinoma. *Vhl* inactivation was directly coupled to expression of a tdTomato reporter within a single allele, allowing accurate visualization of affected cells in their native context and retrieval from the kidney for single-cell RNA sequencing. This strategy uncovered cell type–specific responses to *Vhl* inactivation, defined a proximal tubular cell class with oncogenic potential, and revealed longer term adaptive changes in the renal epithelium and the interstitium. Oncogenic cell tagging also revealed markedly heterogeneous cellular effects including time-limited proliferation and elimination of specific cell types. Overall, this study reports an experimental strategy for understanding oncogenic processes in which cells bearing genetic alterations can be generated in their native context, marked, and analyzed over time. The observed effects of loss of *Vhl* in kidney cells provide insights into VHL tumor suppressor action and development of renal cell carcinoma.

**Significance:**

Single-cell analysis of heterogeneous and dynamic responses to *Vhl* inactivation in the kidney suggests that early events shape the cell type specificity of oncogenesis, providing a focus for mechanistic understanding and therapeutic targeting.

## Introduction

Multiple advances in the molecular and genetic analysis of cancer have transformed understanding of the disease over recent decades (1). However, most work has focused on either the later stages of cancer or on short-term models of aggressive disease. Much less is known about the earliest events and the cellular responses to those events, particularly when progression to cancer is slow. Furthermore, the consequences of oncogenic mutations may be highly context specific, and their occurrence not always associated with neoplastic progression (2). These findings have highlighted a range of new questions as to the interactions and behavior of cells bearing these initiating tumor suppressor and oncogenic mutations that need technologies with accurate cellular and temporal resolution to be properly addressed.

Although advances in technologies for single-cell analyses have the potential to illuminate this gap in knowledge, their application is often restricted by difficulties in the distinction, isolation, and tracking of cells that bear the relevant mutation. For instance, microdissection strategies are dependent on guidance from morphologic abnormalities or downstream markers of the activated pathways that are not necessarily directly coupled to an earlier initiating event. This restriction can be overcome by using an oncogenic cell marking strategy that couples the generation of an oncogenic mutation with the expression of a fluorescent reporter (3–6). Here we have adapted this strategy to develop a new mouse model designed to accurately mark cells that have undergone a Cre-recombinase-induced inactivating mutation even at limiting levels of recombinase activity and have applied this to the study of von Hippel Lindau (*VHL*)–associated oncogenesis.

The *VHL* tumor suppressor gene is a classical tumor suppressor whose biallelic inactivation is associated with a restricted range of tumors, the most important of which is clear cell renal cell carcinoma (ccRCC; refs. 7, 8). *VHL* inactivation is almost universally observed as a truncal event in ccRCC, but is often associated with very long latencies in respect of the development of clinical disease (9). VHL is the recognition component of a ubiquitin E3 ligase complex that targets hypoxia inducible transcription factors (HIF1A and HIF2A) for conditional oxygen-dependent degradation (10), but has also been implicated in a range of other roles including cytoskeletal functions (11), regulation of the extracellular matrix (12), and other growth and inflammatory signaling pathways (13, 14). Though dysregulation of one or more of these processes likely contributes to tumorigenesis, neither the mechanisms of oncogenesis nor those of latency are well understood.

Many studies of the cellular effects of *VHL* inactivation on gene expression have been performed in specific types of cultured cells. These have mostly focused on re-introduction of *VHL* into *VHL*-defective ccRCC cell lines (15), or *VHL* inactivation in previously immortalized or transformed renal cells (16) and do not necessarily capture renal cell–specific effects or progression over time. Other studies have reported effects of *Vhl* inactivation in murine models but have generally not resolved cell-type and temporal effects (17, 18). Recently single-cell sequencing technologies have been applied to the kidney, providing a wealth of data on gene expression patterns, on nephron heterogeneity, and on cellular responses in non-neoplastic disease (19–23).

The system we have developed directly couples inactivation of *Vhl* to the activation of a tdTomato reporter in a single allele, enabling the marking, retrieval, and analysis of *Vhl*-null cells from native kidneys at different time points. We report on the application of this strategy to define the immediate and later consequences of *Vhl* inactivation in mouse kidney at cellular resolution using single-cell transcriptomics and *in situ* methodologies.

## Materials and Methods

### Mice

All experimental procedures were conducted in line with AACR guidelines following approval by the Medical Science Ethical Review Committee of the University of Oxford and authorization under UK Home Office regulations of Animals (Scientific Procedures) Act 1986.

Mice were housed in individually ventilated cages on a 13-hour light/ 11-hour dark cycle with food and water provided *ad libitum*. B6.*Vhl^tm1.1b(tdTomato)Pjr^* (*Vhl^pjr.fl^*) mice were commissioned from Ozgene and generated using goGermline technology (24). *Vhl^tm1jae^* mice (*Vhl^wt/jae.fl^*; ref. 25; RRID:IMSR_JAX:012933) were crossed with *Tg(Pgk1-cre)1Lni* (*Pgk1-Cre*; ref. 26; RRID:IMSR_JAX:020811) to generate a constitutively knocked-out form of the *Vhl^tm1jae^* allele (*Vhl^jae.KO^*). *Tg(Pax8-cre/ERT2)CAmat* (*Pax8-CreERT2*; RRID:IMSR_HAR:9175) mice were obtained via EMMA (27). *Vhl^wt/pjr.fl^; Pax8-CreERT2* (“Control”) and *Vhl^jae.KO/pjr.fl^; Pax8-CreERT2* (“KO”) mice of both sexes were used between 2 and 20 months of age, and all were bred on a C57BL/6J background.

### Nomenclature

Consistent with the biallelic nature of *VHL* tumor suppressor activity, Control refers to the haplo-sufficient *Vhl^wt/pjr.fl^; Pax8-CreERT2* genotype. KO refers to the *Vhl^jae.KO/pjr.fl^; Pax8-CreERT2* genotype. Following tamoxifen administration, induction of Cre recombinase creates chimeric Control and KO mice containing cells that carry either recombined or unrecombined forms of the *Vhl^pjr.fl^* allele. tdTomato-positive cells from KO mice are null (*Vhl^jae.KO/pjr.KO^*) for *Vhl* and are referred to as *Vhl*-null cells. Populations of sorted tdTomato-positive or tdTomato-negative cells from Control mice are referred to as Control positive or Control negative samples, respectively. Populations of sorted tdTomato-positive or tdTomato-negative cells from KO mice are referred to as KO positive or KO negative samples, respectively. Early and late time points refer to 1 to 3 weeks and 4 to 12 months following the first tamoxifen dose, respectively.

### Genotyping and PCR

Genotypes of all experimental mice were reconfirmed at time of harvest. Primer sequences and expected product sizes as follows: V*hl^jae.KO^* : 5′-CTGGTACCCACGAAACTGTC-3′,5′-CTAGGCACCGAGCTTAGAGGTTTGCG-3′; 5′-CTGACTTCCACTGATGCTTGTCACAG-3′ (260 bp for *Vhl^wt^, *240 bp for *Vhl^jae.KO^* multiplex products); *Vhl^pjr.fl^* : 5′- GGTGCTAATTGAAGGAAGCTACTG-3′ and 5′- CTCCTCCGAGGACAACAACATG-3′ (1067 bp); *Vhl^pjr.KO^* : 5′-GCTTTGAGAATTTTGGGCAGTTG-3′ and 5′- TGAAGTTAGTAGCTCCGCTTCCAC-3′ (521 bp); *Pax8-CreERT2* : 5′- CGGTCGATGCAACGAGTGATGAGG-3′, 5′-CCAGAGACGGAAATCCATCGCTCG-3′, 5′-CTCATACCAGTTCGCACAGGCGGC-3′ and 5′- CCGCTAGCACTCACGTTGGTAGGC-3′ (300 and 600 bp multiplex products).

### Tamoxifen administration

Tamoxifen solution was prepared by diluting a 25% (w/v) solution of tamoxifen (Sigma, T5648) in ethanol to 20 mg/mL in corn oil (Sigma, C8267). Following pilot experiments with different tamoxifen doses, we chose a 5 × 2 mg tamoxifen dosing regimen that induced sufficient levels of recombination in a consistent manner and created chimeric mice in which recombined and unrecombined cells could be compared; this regimen was in line with methodology used by others for the *Pax8-CreERT2* allele (27, 28). Five consecutive daily doses of 2 mg tamoxifen were administered to Control and KO mice >20 g by oral gavage using sterile, disposable plastic feeding tubes (Instech, FTP-20–38). The median age of mice at tamoxifen administration was 11 weeks (interquartile range: 9–18 weeks). We did not observe any correlation between the age at which the mice were dosed and either the levels of recombination induced or in the consequences of *Vhl* inactivation.

### Immunohistochemistry

#### Tissue preparation

IHC was performed on kidneys from all experimental mice following either immersion-fixation or perfusion-fixation depending on downstream analyses. Immersion fixation was used when kidney tissue from the same mouse was also used for immunoblotting or single-cell RNA sequencing (scRNA-seq). Mice were euthanized with terminal isoflurane anesthesia and flushed via the aorta with 1× PBS pH 7.4 (Gibco, 70011) to clear blood from tissues. For immersion fixation, kidneys were bisected and fixed in 10% neutral-buffered formalin (NBF; Sigma, HT501128) with rocking for 24 hours at room temperature. For perfusion fixation, flushing with PBS was followed by perfusion of 4% (w/v) paraformaldehyde (Sigma, P1213) in 1× PBS (Gibco) pH 7.4 at room temperature and kidneys processed as for immersion fixation. For frozen sections, kidneys were allowed to sink in 30% (w/v) sucrose in PBS before freezing in optimal cutting temperature compound (OCT; Thermo Fisher Scientific, 12678646) over dry ice. For formalin-fixed paraffin-embedded (FFPE) sections, kidneys were dehydrated through a graded ethanol series (70% to 100%) and xylene before paraffin embedding. OCT-embedded tissues were cut to 8 μm sections at −28°C with a Bright OTF5000/LS-001 cryostat. Sections were mounted on poly-lysine coated slides and air-dried at room temperature before storage at −80°C or use in Oil Red O (ORO) staining. FFPE tissues were cut to 4 μm sections on a Thermo Microm HM 355S Microtome using MB35 Premier Blades. Sections were floated on warm distilled water and mounted on poly-lysine coated slides (Thermo Fisher Scientific, 10149870). Slides were dried for at least 3 hours at 37°C before IHC.

#### Single-label IHC

FFPE sections were deparaffinized with xylene and ethanol and rehydrated with double-distilled water. For tdTomato and CD45 IHC, sections were subjected to heat-induced epitope retrieval (HIER) with 10 mmol/L Tris, 1 mmol/L EDTA, pH 9.0 in a steamer (at 95°C) for 20 minutes. Slides were blocked for endogenous peroxidase activity using Dako Peroxidase Blocking solution (Agilent, S2023) for 10 minutes at room temperature and nonspecific protein binding using 5% (w/v) bovine serum albumin (BSA; Sigma, 5482) in 1× TBST [50 mmol/L Tris, 31.6 mmol/L NaCl, 0.1% (v/v) Tween-20; pH 8.4] for 40 minutes at room temperature before incubation with primary antibody diluted in Dako Antibody Diluent Solution (Agilent, S3022) overnight at 4°C (tdTomato—Rockland, 600–401–379, RRID:AB_2209751, 1:1,000; CD45—Cell Signaling Technology, 70257, RRID:AB_2799780, 1:200). Sections were washed in 1× TBST. Signal was detected with the Dako Envision system (Agilent, K4003) with a diaminobenzidine (DAB) exposure time of 10 minutes for tdTomato and 1.5 minutes for CD45, and counterstained with modified Harris Hematoxylin (Thermo Fisher Scientific, 72711) differentiated for 10 seconds in 0.25% HCl in 70% ethanol. Hematoxylin staining was blued by immersion in 0.06% (w/v) NH_4_OH in water for 30 seconds. Slides were dehydrated and mounted with DPX mountant (Merck, 06522).

#### tdTomato-Ki67 dual IHC

tdTomato was visualized using the standard Envision IHC protocol as detailed above, with a 3 minutes DAB (brown) exposure to generate a first signal of lower intensity. Peroxidase activity was neutralized by incubating slides in 3% (v/v) H_2_O_2_ (Merck, H1009) in 1× PBS, and a second round of HIER performed with Dako Antigen Retrieval Solution (Agilent, S1699) in a pressure cooker (at 120°C) for 12 minutes. Slides were then blocked for avidin, biotin, and protein binding (Abcam, ab64212; Agilent, X090930–2). Ki67 was detected using a biotinylated Rabbit anti-Ki67 biotin-conjugated antibody (Life Technologies, 13569882, RRID:AB_2572794) at room temperature for 2 hours, before being recognized by streptavidin-HRP provided in the BrdU Detection Kit II (BD, 551321) at room temperature for 1 hour. Staining was visualized with Vector VIP (purple) substrate (Vector, SK4605) with a 1.5 minutes exposure time. Sections were counterstained and mounted as described previously.

#### Trichrome staining

Trichrome staining was performed on FFPE sections using the Trichrome Stain (Connective Tissue Stain) Kit (Abcam, ab150686) according to manufacturer's instructions. Briefly, FFPE sections were deparaffinized and rehydrated as described before treatment with preheated Bouin's fluid at 60°C for 1 hour. Slides were washed in tap water and stained with Weigert's Iron Hematoxylin for 5 minutes at room temperature and Biebrich Scarlet/acid fuchsin solution for 15 minutes at room temperature. Staining was differentiated by incubation with phosphomolybdic/phosphotungstic acid for 15 minutes at room temperature; this removed the “red” staining from collagen in the tissue section. Collagen was then stained with Aniline blue solution for 15 minutes at room temperature differentiated with 0.1% acetic acid solution for 3 minutes at room temperature. Slides were dehydrated in a graded series of ethanol and xylene as described before and mounted in DPX.

#### Hematoxylin and eosin staining

FFPE sections were deparaffinized and rehydrated and stained with hematoxylin as described above. Sections were rinsed twice each in 90% and 100% ethanol and then incubated in Shandon Alcoholic Eosin Y solution (Thermo Fisher Scientific, 6766008) for 3 minutes at room temperature before washing in 100% ethanol, clearing with xylene, and mounting with DPX.

#### Periodic acid-Schiff staining

FFPE sections were deparaffinized and rehydrated as described before incubation in 0.5% periodic acid solution (Sigma, 100482) for 15 minutes at room temperature. Sections were washed in double-distilled water before incubation in Schiff's reagent (Sigma, 3952016) for 1.5 minutes in a microwave and rinsing under running hot tap water for 3 minutes. Sections were then counterstained with Harris hematoxylin for 90 seconds without differentiation and bluing, dehydrated and mounted with DPX as described above.

#### Oil Red O staining

ORO staining was performed with the Oil Red O Staining Kit (Abcam, 150678) as per manufacturer's instructions. Briefly, OCT-embedded frozen sections were brought to room temperature for 10 minutes and then washed in PBS for 10 minutes. Sections were then incubated in 100% (v/v) propylene glycol solution for 10 minutes at room temperature, followed by incubation in preheated Oil Red solution for 20 minutes at 60°C. The stain was then differentiated in 85% (v/v) propylene glycol in double-distilled water for 1 minute at room temperature, before rinsing in double-distilled water. Sections were then counterstained with Harris hematoxylin without differentiation and bluing and were directly mounted using VectaShield Vibrance Antifade mountant (Vector, H170010).

#### Microscopy

IHC sections were scanned with a Hamamatsu NanoZoomer S210 slide scanner at ×40 magnification and viewed on Hamamatsu NDP.view2 software.

#### Quantification

Quantification of the proportion of cells staining positive for tdTomato or Ki67 by IHC was performed using the HALO Image Analysis Software v3.5 and v3.6 (Indica Lab). Kidney sections were annotated manually into cortex, outer medulla, inner medulla, and papilla areas. Nuclei were detected on the basis of hematoxylin staining using the HALO AI v3.6 Nuclei Seq algorithm with detected nuclei <15 and >200 μm^2^ excluded from analysis. The red–green–blue (RGB) image of the kidney section was computationally deconvoluted into individual contributions from hematoxylin, DAB, and/or VIP based on known colorimetric properties of the three stains. Cells were recorded as tdTomato-positive if the tdTomato (DAB) signal passed intensity threshold in detected nuclei and in a 2 μm perimeter drawn around each nucleus. Cells were scored as positive for Ki67 if the signal (VIP) passed threshold in the nucleus. Identical analysis parameters were used for all slides stained for the same targets. One entire section was analyzed per mouse, with an average of 95,503, 38,293, 49,258, and 16,330 cells counted in the cortex, outer medulla, inner medulla, and papilla in every section, respectively.

### RNA *in situ* hybridization

RNA *in situ* hybridization was performed on 4 μm FFPE kidney sections cut on the previous day and baked at 60°C for 1 hour using either RNAscope 2.5 HD Assay – BROWN (ACD BioSciences, 322300) for single assays, or RNAscope 2.5 HD Duplex Detection Kit (ACD BioSciences, 322430) for dual assays, according to manufacturer's instructions. Probes used were: *Apoc3* (catalog no. 518591), *Defb19* (catalog no. 1245931-C1), *Neat1* (catalog no. 440351), and *Fxyd2* (catalog no. 572631-C2). For single assay, sections were counterstained and mounted as for IHC.

### Tissue dissociation

Kidneys were dissociated using the Multi-Tissue Dissociation Kit 2 (Miltenyi, 130–110–203). Briefly, the renal capsule was removed, and the tissue macerated for 5 minutes on a bed of ice. The macerated kidney was then transferred to 1.45 mL Buffer X, 30 μL of Enzyme D, 15 μL of Enzyme P, 15 μL of Buffer Y, and 6 μL of Enzyme A, all prepared according to the manufacturer's instructions. The tube was then agitated under water at 37°C in a shaking incubator at 150 rpm for 30 minutes. Dissociation was stopped by the addition of 150 μL FBS (Sigma, F7524), and resuspension in 9 mL of RPMI-1640 medium (Merck, R0883). Undigested tissue was removed using a 40 μm cell strainer and the filtrate centrifuged (300 × *g* for 10 minutes at 4°C). Erythrocytes were eliminated by resuspending dissociated cells in 3 mL of 1× RBC Lysis Buffer (Miltenyi, 130–094–183) prepared in deionized water for 2 minutes at room temperature. Cells were centrifuged (300 × *g* for 5 minutes at 4°C) and resuspended in ice-cold D-PBS before being counted on a Thermo Fisher Scientific Countess II machine for total yield and viability.

### FACS

Dissociated cells were resuspended in 10% FBS, 2 mmol/L EDTA, in D-PBS (Thermo Fisher Scientific, 14190144) to a concentration of 5 million live cells per milliliter. DAPI (Sigma, D9542) was added to stain for viability. Cells were sorted using a BD Aria Fusion Cell Sorter. tdTomato was excited with a 561 nm laser and fluorescence detected with a 582/15 band pass filter. DAPI was excited with a 405 nm laser and fluorescence detected with a 450/40 band pass filter. Live, single, tdTomato-positive and -negative cells were collected in FBS-coated polypropylene tubes and pelleted by centrifugation at 300 × *g* for 10 minutes. Cells were counted again for yield and viability and processed for immunoblotting, DNA extraction, or scRNA-seq.

### Immunoblotting

Protein extraction was performed by solubilizing cell pellets recovered from FACS and frozen over dry ice in Urea SDS buffer (7 mol/L Urea, 10 mmol/L Tris, 1% SDS, 10% glycerol, pH 7.4), adding Laemmli Sample buffer to 1.5× (v/v) and a final concentration of 7,000 cells/μL, and then boiling at 95°C for 5 minutes. Immunoblotting was performed as described previously (29). Briefly, proteins were separated by SDS-PAGE, transferred to polyvinylidene difluoride membrane (Immobilon-P; Millipore, IPVH00010), and blocked in 4% (w/v) fat free milk in 1x PBS with 0.1% Tween 20. Primary antibodies: tdTomato (Rockland 600–401–379; RRID:AB_2209751), HIF1A (Cayman, 10009269; RRID:AB_999557), and HIF2A (PM9, custom-made and characterized previously; ref. 30) were diluted in blocking buffer (1:1,000). HRP-conjugated secondary antibodies (Agilent, P0448; RRID:AB_2617138) and chemiluminescence substrate (West Dura; Thermo Fisher Scientific, 34076) were used to visualize proteins, using a ChemiDoc XRS+ imaging system (Bio-Rad). After immunoblot analysis, membranes were stained with Coomassie brilliant blue to visualize separated proteins, and this was used as a reference of sample loading.

### Single-cell GEM and library preparation

Sorted cells were prepared into single-cell droplets using the Chromium Next GEM Single-Cell Kit. A total of 20,000 live cells were loaded per sample on separate Chromium Next GEM Chip G (10X Genomics, PN-1000127) channels. cDNA clean-up, amplification, and adaptor ligation were performed with the Chromium Next GEM Single-Cell 3′ Kit v3.1 (10X Genomics, PN-1000268). Twelve reaction cycles were used for cDNA amplification. cDNA yield was quantified using the High Sensitivity D1000 ScreenTape Assay (Agilent, 5067–5584 and 5067–5585) to optimize the number of reaction cycles for library preparation. Single-cell sequencing libraries were prepared using Dual Index Kit TT Set A (10X Genomics, PN-1000215) sequence indices. Prepared libraries were quantified with KAPA Library Quantification Kit (Roche, KK4824) following the manufacturer's protocol.

### Sequencing

Single-cell libraries were sequenced on Illumina NextSeq 2000 sequencing system with P3 200 cycle (Illumina, 20040560) reagents. Equimolar pools of up to six sample libraries were prepared and diluted to 650 pmol/L and mixed with 1% PhiX Control v3 DNA (Illumina, FC-110–3001) in RSB buffer to a final volume of 25 μL. Sequencing was performed and preliminary sequencing results (bcl files) and FASTQ files were generated with the DRAGEN FASTQ Generation 3.8.4 or the DRAGEN BCL convert workflow optimized for Single-Cell RNA Library Kit 1 Library Prep Kit, and the Single-Cell RNA Index Adaptors 1-B Index Adaptor Kit, with 28 and 152 Read 1 and 2 cycles, respectively.

### Single-cell sequencing data processing and quality control

The mm10 reference genome was customized and the starting reference fastq files and GTF files came from refdata-gex-mm10–2020-A.tar.gz and are available for download at https://www.10xgenomics.com/support/single-cell-gene-expression or https://cf.10xgenomics.com/supp/cell-exp/refdata-gex-mm10–2020-A.tar.gz. The transcript sequence for *Vhl^pjr.KO^* that included the tdTomato sequence was then added manually to the fasta reference genome file and annotation for tdTomato transcript was added manually to the gtf file. The cellranger 6.0.1 mkref function was used to build the custom reference genome and reads taken from FASTQ files generated for each sample were aligned to the custom reference genome by CellRanger (version 6.1.1; https://support.10xgenomics.com/single-cell-gene-expression/software/pipelines/latest/what-is-cell-ranger) using default parameter sample by sample. After aligning, for each read pair, cell barcodes and unique molecular identifiers (UMI) were obtained from Read 1 and read counts per feature were obtained from Read 2. Only those UMIs that could be linked to a valid cell barcode and a gene exon region were included to create the cell by gene count matrix. Reads from *Vhl* exon 1 were excluded from analysis to prevent ambiguous alignment. Cells were subjected to the following filters: detected genes >200, fraction of mitochondrially-encoded reads <0.5, and detected genes <3× median for each sample. The threshold for mitochondrially encoded reads was set to this value in line with published kidney scRNA-seq studies to account for the high mitochondrial content in the renal tubular epithelium (RTE) cells (20). Information and quality control metrics for all sequenced samples are provided in Supplementary Table S1. Cells from different samples and sequencing runs were then aggregated, which sometimes required manual renaming of duplicate cell “names.” Downstream analysis was conducted using the R package Seurat 4.0.3: Read counts were log-normalized and scaled, principal component analysis (PCA) was run using the scaled data and Uniform Manifold Approximation and Projection (UMAP) was generated using the top 30 PCs.

### Batch correction and dimension reduction

Gene expression in RTE cells, and proximal tubule (PT) cells in particular, has been reported to be highly sex-specific (23, 31, 32). To account for this difference, batch correction was performed based on sex using Seurat following “Tips for integrating large datasets” from the Vignettes of Seurat. Samples were split by sex. Briefly, for each sex, read counts were log-normalized and the top 2,000 varying features were identified and analyzed using the FindVariableFeatures command. Next, features were selected for downstream integration using SelectIntegrationFeatures and for each sex, normalized read counts were scaled using these selected features and PCA was run afterwards. Then anchoring features between the two sexes were calculated using FindIntegrationAnchors functions with parameter “reduction = ’rpca’,” which used reciprocal PCA (top 30 PCs) to identify an effective space in which to find anchors. Finally, samples of the two sexes were integrated with the IntegrateData function using the identified anchoring features. For the integrated data, standard Seurat workflow was performed: Expression of these genes was scaled to have a mean at 0 and SD of 1 using the ScaleData function. PCs were analyzed for the expression of these genes using the RunPCA function. The first 30 components were then included for shared nearest neighbor modularity optimization with the Louvain algorithm using the FindClusters function and resolution set to 1. Finally, UMAP dimension reduction was performed using the RunUMAP function with default parameters and 30 dimensions.

### Inferring cell types in scRNA-seq

Lists of genes differentially expressed across renal cell types in wild-type C57BL/6J mice were obtained from previous work (20–23). For each cell type described in these papers, marker genes were selected as those with log_2_-fold change (compared with other cells in the dataset) >1.0, and which were expressed in >50% of cells they marked, and <20% of the cells they did not mark. Separate markers were listed for male and female proximal tubular S2 and S3 cells, based on reported sex-specific expression of markers (23). The four papers to which we referred did not always divide renal cell types in the same manner. Conflicts were resolved with reference to histologic and anatomical literature on the kidney (33, 34). Marker gene lists were prepared for the following cell types: three segments of the proximal tubule S1, S2, and S3; loop of henle epithelium, distal convoluted tubule, principal and intercalated cells of the collecting duct, parietal cells, podocytes, endothelial cells, fibroblasts, vascular smooth muscle cells, pericytes, macrophages, monocytes, neutrophils, NK cells, granulocytes, B lymphocytes, and T lymphocytes and are provided in Supplementary Table S1.

Genes present in this list but not detected in any cells in our dataset were excluded from analysis. Marker gene lists were then converted into “gene module scores” using Seurat's AddModuleScore (35) function, with 100 bins and 50 control genes used per bin. Individual cells were assigned the cell type that attained the highest module score. Only one of the four papers to which we referred (23) reported data from mice of both sexes and found sex-specific cell type marker gene expression in PT S2 and S3 cells. Accordingly, sex-specific modules for S2 and S3 PT cells were used in this assessment for male and female cells.

### Identifying anticorrelated gene sets and annotating PT classes

The top 1,200 varying genes in PT cells from tdTomato-positive and -negative cells from Control mice were identified by calculating the variance in normalized expression for every detected gene across cells using the var function in R. Pairwise Spearman correlation coefficients were calculated for each combination of genes, resulting in a 1,200 × 1,200 correlation matrix. Hierarchical clustering was performed on this matrix using ‘hclust’, and heatmap plotted using the ‘heatmap.2’ function in R. Two “sets” of anticorrelated genes were visually distinguished, and genes contributing to each set were identified from the dendrogram using “cutree.”

Each gene set was converted to a module and used to score cells using the AddModuleScore function with 100 bins and 50 controls per bin. PT Class A cells were identified as those with Module A expression score >0.15 and the others were identified as PT Class B cells.

### Differential expression testing

Differential expression (DE) testing was performed for various combinations of cells using the FindMarkers function of Seurat in R using Wilcoxon test and Bonferroni correction for multiple testing. Normalized, unscaled expression was used for calculating log_2_-fold changes between conditions. Genes were considered regulated if robustly altered (|log_2_-fold change| > 0.25), and statistically significant (adjusted *P* value < 0.01). DE testing was performed in individual cell identities if >100 cells of that identity were present in each condition. All types and classes of PT cells, and intercalated cells of the collecting duct (CDIC) cells passed this threshold in tdTomato-positive cells. In addition to these identities, macrophage, monocyte, neutrophil, NK cell, granulocyte, B lymphocyte, and T lymphocyte cell types passed this threshold in tdTomato-negative cells.

#### Genes regulated by *Vhl* at the early time point

DE was first tested in all tdTomato-positive cells from KO mice versus Control mice harvested at the early time point, and genes that were regulated were identified. The same analysis was then performed comparing tdTomato-negative cells in KO mice versus Control mice at the early time point, and genes regulated identified. Genes regulated in tdTomato-positive cells, but not regulated in the same direction in tdTomato-negative cells, were considered to be specifically regulated by *Vhl* inactivation at the early time point.

#### Genes regulated with time in *Vhl*-null cells

DE was first tested in tdTomato-positive cells from KO mice at the late versus early time points, and genes that were regulated were identified. The same analysis was then performed comparing tdTomato-positive cells in Control mice at the late versus early time points, and genes that were regulated were identified. Genes regulated in cells from KO mice, but not regulated in the same direction in cells from Control mice, were considered to be regulated over time specifically in *Vhl*-null cells.

#### Comparing *Vhl*-dependent gene expression across cell identities

DE testing was performed as described above but for subsets of cells of individual cell identities, and a union list of upregulated and downregulated genes across all identities was prepared. Log_2_-fold changes for these genes were calculated for cells of each identity in tdTomato-positive cells from KO versus Control mice at the early time point. PCA was performed on these changes using the prcomp function in R, after scaling and centering data.

#### Cell type–specific *Vhl*-regulated genes

DE testing was performed as described above, but for subsets of cells of individual cell identities. CDIC-specific or PT S3-specific genes were identified as those that were regulated in CDICs or PT S3, respectively, but in no other cell identity in the same direction, and for which Δ|log_2_-fold change| > 0.2 in CDICs or PT S3, respectively, compared with all other identities. PT Class A specific genes were identified as those that were significantly regulated in PT Class A cells but not PT Class B, and for whom Δ|log_2_-fold change| > 0.2 in PT Class A compared with PT Class B.

#### Genes regulated in renal interstitial cells

DE testing was performed on tdTomato-negative cells from KO *v*ersus Control mice at the late time point for cells of the following cell types separately: macrophage, monocyte, neutrophil, NK cell, granulocyte, B lymphocyte, and T lymphocyte. GO term annotation was performed for genes upregulated in each cell type using gProfiler (https://biit.cs.ut.ee/gprofiler/gost; ref. 36).

### Reproducibility between replicates

Log_2_-fold changes were calculated between individual KO positive samples versus all sex-matched Control positive samples at the early time point, for *Vhl*-regulated genes identified as above. Spearman correlation coefficients were calculated using the cor function in R for log_2_-fold changes for matched genes between individual KO samples and plotted as a heat map.

### Gene set enrichment analysis

Gene set enrichment analysis (GSEA) was performed using the fgsea package in R, and barcode plots created using the PlotEnrichment function in R. Genes regulated in *Vhl*-null cells at the late versus early time points were ranked according to log_2_-fold change. Genes identified as regulated specifically in all *Vhl*-null cells at the early time point were listed as “early upregulated genes.” Mouse orthologs of known HIF target genes (see below) were listed as “Known HIF targets.” Genes regulated in *Vhl*-null PT like cells compared with *Vhl*-null PT cells were ranked according to log_2_-fold change. Cell-type marker gene lists prepared above were used as gene sets to test enrichment of particular cell type marker genes.

### Definition of HIF target genes

Canonical HIF target genes have been previously defined in six human cancer cell lines based on HIF genome binding (chromatin immunoprecipitation sequencing) and hypoxic regulation (RNA-seq; ref. 37). Briefly, for each cell line, a HIF target gene was defined as being induced by hypoxia and having a HIF1 (HIF1A and HIF1B) and/or HIF2 (HIF2A and HIF1B) binding site in proximity to that gene (gene must be in the three closest transcriptional start sites to the binding site). Merging the HIF target genes identified in each cell line provides a list of 1,301 known, *bona fide* HIF target genes; encompassing both cell type–conserved and cell type–specific targets. Mouse orthologs for these genes were obtained using the babelgene package in R. 1,226 genes were obtained and are given in Supplementary Table S1.

### Definition of ccRCC prognosis genes

Pathology data in The Human Protein Atlas (https://www.proteinatlas.org/, RRID:SCR_006710) was searched on July 27, 2023, for genes specifically regulated in PT Class A but not PT Class B cells to determine if any were identified as prognostic markers for renal cancer and relevant Kaplan–Meier plots for relevant genes exported.

### Definition of genes upregulated in ccRCC

A recent scRNA-seq study on human ccRCC samples reported genes upregulated in ccRCC tumor cells compared with normal PT cells (38). Genes that were upregulated in at least 25 of the 30 tumors analyzed were considered to be “upregulated in ccRCC” in our analysis. Mouse orthologs for these genes were obtained using the babelgene package in R. These were compared with the list of HIF target genes as defined above to identify HIF-driven and non-HIF driven genes upregulated in ccRCC (Supplementary Table S1). These were then converted to modules and expression scored using the AddModuleScore (35) function in R, with 100 bins and 50 control genes per bin.

### Identification of PT like cells

Cell clusters were determined after batch correction for sex using Seurat for KO positive and Control positive samples harvested at the early or late time points. Clusters in which >80% cells were of S1, S2, or S3 PT cell type were considered “PT clusters.” Cells assigned to non-PT cell types, which were present within PT-clusters were considered to be PT like cells.

### Statistical analysis

Differences in retention of tdTomato-positive cells in Control or KO mice over time were analyzed by comparing linear fitted models using the lm function in R. *t* testing between intercept and slope of fitted models was used to assess significance of differences in initial recombination and survival over time, respectively. Associated *P* values were adjusted by Bonferroni correction for multiple testing.

For comparison of means, data were first subjected to D'Agostino-Pearson omnibus normality test. If the normality test was passed, differences between groups were tested by one-way ANOVA (data grouped by one variable) or two-way ANOVA (data grouped by two variables) with Holm–Šídák multiple testing correction. If normality was not passed, differences were tested by Mann–Whitney test or Kruskal–Wallis test with Dunn multiple testing correction.

For DE testing, *P* values calculated by Wilcoxon tests by the FindMarkers function were adjusted by Bonferroni correction for multiple testing.

### Data visualization

Single-cell sequencing data were visualized either with in-built functions of heatmap.2 or fgsea, or using the ggplot2 and cowplot packages in R (versions 4.2.3 and 4.3.2). Statistical analyses and relevant plots were also generated in Prism 10.

### Data availability

The data generated in this study are publicly available in Gene Expression Omnibus (GEO) at GSE253168. All other raw data generated in this study are available upon request from the corresponding author.

## Results

### A novel reporter of *in vivo Vhl* inactivation in mice

**Figure 1.**
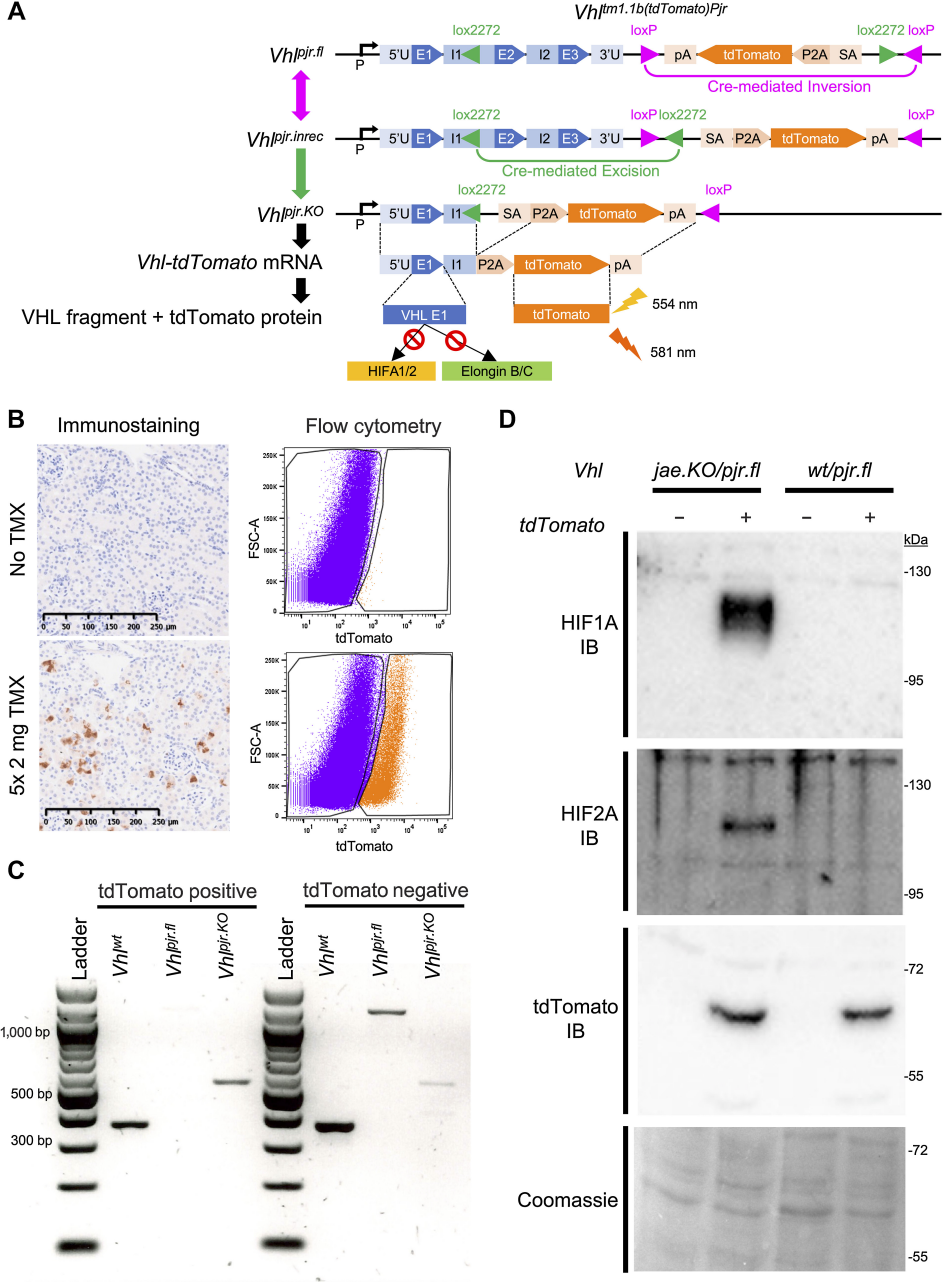
A novel reporter model for *Vhl* inactivation in the mouse kidney. **A,** Design and recombination of the cell marking conditional *Vhl^pjr^* allele. Double and single arrows indicate reversible and irreversible processes, respectively. *Vhl^pjr.fl^, Vhl^pjr.inrec^*, and *Vhl^pjr.KO^* refer to “floxed,” “incompletely recombined,” and “knockout” forms of the *Vhl^pjr^* allele. P, *Vhl* promoter; U, untranslated region; E, *Vhl* exon; I, *Vhl* intron; pA, polyadenylation site; P2A, porcine teschovirus 2A peptide; SA, splice acceptor. Dashed lines, spliced and translated regions; lightning symbols, excitation and emission wavelengths for tdTomato fluorescence. Red stop sign indicates no interaction between VHL exon 1 fragment and HIFA-1/2 or Elongin B/C. **B,** Representative tdTomato IHC counterstained with hematoxylin in kidney sections and tdTomato fluorescence-based flow cytometry on renal cells from *Vhl^wt/pjr.fl^; Pax8-CreERT2* mice untreated (top) or given 5 × 2 mg tamoxifen (TMX; bottom) and harvested at the early time point. Scale bar, 250 μm. Magnification, ×20. FACS gates are shown. **C,** Gel electrophoresis of genomic PCR for *Vhl^wt^, Vhl^pjr.fl^*, and *Vhl^pjr.KO^* alleles performed on FAC-sorted tdTomato-positive (left) or tdTomato-negative (right) cells from kidneys of *Vhl^wt/pjr.fl^; Pax8-CreERT2* mice given tamoxifen and harvested at the early time point. **D,** Representative immunoblots (IB) for HIF1A, HIF2A, or tdTomato protein in tdTomato-negative (−) or tdTomato-positive (+) cells sorted by flow cytometry from dissociated kidneys of *Vhl^jae.KO/pjr.fl^* or *Vhl^wt/pjr.fl^ Pax8-CreERT2* mice given 5 × 2 mg tamoxifen and harvested at the early time point (*n* = 3 per genotype).

We designed a conditionally inactivated allele (*Vhl^tm1.1b(tdTomato)Pjr^*; termed *Vhl^pjr.fl^*) that marks deletion of exons 2 and 3 of *Vhl* by knock-in of a tdTomato reporter gene at the endogenous *Vhl* locus (Fig. 1A; ref. 39). Cre-mediated recombination occurs in two steps: a reversible inversion between two antiparallel *loxP* sites orients the tdTomato cassette sense and downstream to *Vhl* and re-orients a pair of *lox2272* sites to a parallel conformation (*Vhl^pjr.inrec^*). This allows an irreversible recombination between the *lox2272* sites that excises *Vhl* exons 2–3 and the 3′-UTR, thereby positioning the tdTomato reporter downstream of the endogenous *Vhl* promoter and exon 1 fragment (*Vhl^pjr.KO^*). A splice-acceptor allows cotranscription of tdTomato with *Vhl* exon 1 and a porcine teschovirus 2A sequence uncouples their translation. The product from *Vhl* exon 1 lacks residues necessary for interaction with VHL binding partners and substrates (10, 40) and hence is expected to be nonfunctional. Thus, this allele structurally links *Vhl* inactivation with the expression of a fluorescent tdTomato reporter.

We combined the *Vhl^pjr.fl^* allele with either a wild-type or a constitutively inactivated (25) *Vhl* allele (termed *Vhl^jae.KO^*) to create inducible monoallelic or biallelic *Vhl* inactivation, respectively. We used a *Pax8*-driven tamoxifen-inducible Cre allele (*Pax8-CreERT2*), the expression of which is restricted to the RTE (27). Both genotypes were treated with 5 × 2 mg tamoxifen and harvested early (1–3 weeks) or late (4–12 months) after the first dose of tamoxifen.

tdTomato-positive cells could be identified by IHC in kidney sections and *ex vivo* by flow cytometry in cells dissociated from kidneys from *Vhl^wt/pjr.fl^; Pax8-CreERT2* mice given 5 × 2 mg tamoxifen and harvested at the early time point (Fig. 1B). We confirmed by genomic PCR of flow-sorted cells that tdTomato-positive cells had recombined *Vhl^pjr^*, and only carried the recombined *Vhl^pjr.KO^* allele. In contrast, the unrecombined *Vhl^pjr.fl^* allele could be detected in tdTomato-negative sorted cells (Fig. 1C). The recombined allele was sometimes detected in tdTomato-negative cells too, albeit at a much lower levels than in tdTomato-positive cells (Fig. 1C). This suggested an imperfection in negative sorting, possibly arising from relatively low expression of tdTomato driven by the endogenous *Vhl* promoter. Thus, although tdTomato negativity cannot completely exclude the possibility of *Vhl* recombination, tdTomato-positivity accurately marks *Vhl* recombination.

To model conditional biallelic *Vhl* inactivation, we crossed *Vhl^pjr.fl^* with a constitutively inactivated *Vhl* allele (termed *Vhl^jae.KO^*) and *Pax8-CreERT2*, to create *Vhl^jae.KO/pjr.fl^; Pax8-CreERT2* mice. Because HIF1A and HIF2A should be stabilized as a consequence of *Vhl* inactivation, we performed immunoblotting for these proteins in sorted renal cells from *Vhl^jae.KO/pjr.fl^* and *Vhl^wt/pjr.fl^* mice harvested at the early time point. Both proteins were stabilized only in tdTomato-positive cells from *Vhl^jae.KO/pjr.fl^* mice, not in those from *Vhl^wt/pjr.fl^* mice, nor in any tdTomato-negative population (Fig. 1D). This confirmed that marked cells in *Vhl^jae.KO/pjr.fl^* mice were functionally *Vhl*-null, and that tdTomato-positive cells in *Vhl^wt/pjr.fl^* mice were *Vhl*-haplosufficient, consistent with the designation of *VHL* as a classical tumor suppressor needing biallelic inactivation to promote oncogenesis. Because our primary aim was to assess the consequence of somatic inactivation of the second *Vhl* allele as occurs in *VHL*-associated oncogenesis, the *Vhl^wt/pjr.fl^; Pax8-CreERT2* and *Vhl^jae.KO/pjr.fl^; Pax-CreERT2* genotypes are henceforth referred to as “Control” and “KO” respectively, for simplicity (see Materials and Methods).

### scRNA-seq on flow-sorted renal cells

The early *in vivo* effects of biallelic *Vhl* inactivation in different cells of the RTE have not been described previously. Using scRNA-seq, we first analyzed changes in gene expression at the early time point in recombined cells or their unrecombined neighbors from Control and KO mice. Following quality control (see Materials and Methods), we obtained data from 232,101 high-quality cells across 15 samples from 8 mice of both sexes, with an average of 15,473 ± 5731 (SD) cells, 3,265 ± 1490 (SD) transcripts per cell, which covered 1,281 ± 380 (SD) genes per cell per sample. Cell type was determined by the expression of renal cell type specific marker genes, as defined in published scRNA-seq studies (see Materials and Methods; refs. 20–23). Unbiased dimension reduction and clustering was performed, and each cell was labelled according to its inferred cell type. We confirmed that cells expressing a set of cell type markers also shared global transcriptomic profiles by noting that in 36 of the 44 clusters resolved using dimension reduction analyses of internal data, >80% of cells within the cluster were from one or two closely related cell types as defined by reference to external published data (Supplementary Fig. S1A; refs. 20–23).

**Figure 2.**
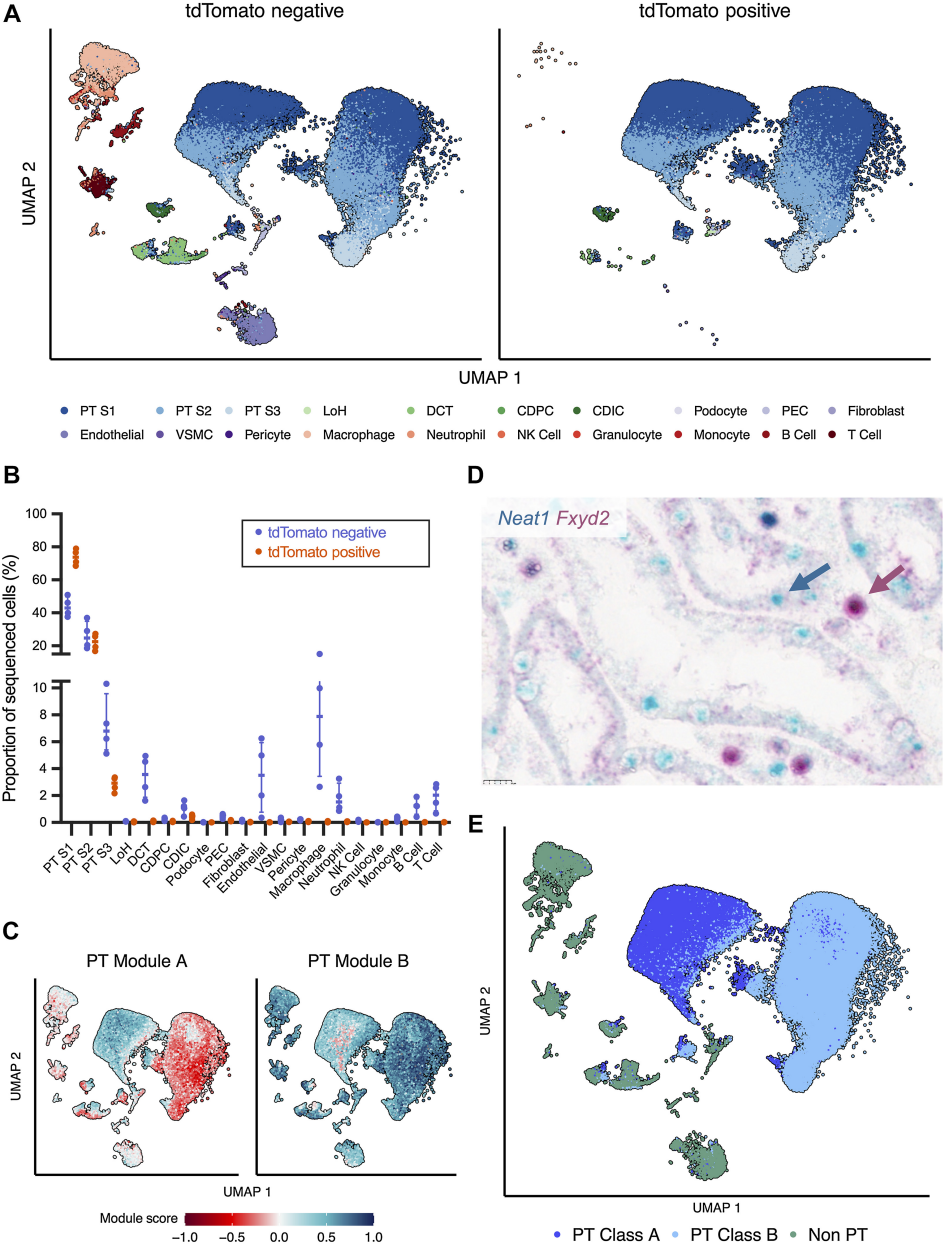
scRNA-seq on flow-sorted renal cells. **A,** UMAP plot of tdTomato-negative (left) or tdTomato-positive cells (right) from kidneys of Control mice harvested at the early time point. Cells are colored by inferred cell type. LoH, loop of Henle; DCT, distal convoluted tubule; CD, collecting duct; PC, principal cell; IC, intercalated; PEC, parietal epithelial cell; VSMC, vascular smooth muscle cell; NK, natural killer cell. **B,** Proportion of sequenced cells inferred to be of each cell type in tdTomato-positive or tdTomato-negative populations from kidneys of Control mice harvested at the early time point. Median and interquartile range plotted. **C,** UMAP plot depicting expression of PT Module A (left) and PT Module B (right) genes in PT cells from Control mice. **D,** Representative *in situ* RNA hybridization exhibiting spatially distinct expression of *Neat1* (blue) and *Fxyd2* (red) mRNA in FFPE kidney cortex from Control mice harvested at the early time point. Scale bar, 10 μm. Magnification, ×40. **E,** UMAP plot depicting cells from Control mice at the early time point. Cells are colored by assigned PT Class. **A–E,** scRNA-seq data shown for *n* = 3 female (3F) and *n* = 1 male (1M) Control mice.

A range of renal cell types were recovered in tdTomato-negative cells from kidneys of Control mice, including epithelial proximal tubular, distal tubular, and collecting duct cells; nonepithelial interstitial and vascular cells and multiple immune cells subsets (Fig. 2A and B). By comparison tdTomato-positive cells from the same mice contained mostly PT and CDIC cells, consistent with the reported tubular epithelial restriction of *Pax8-CreERT2* (27). The PT and CDIC cells in both tdTomato-positive and -negative cells from kidneys of Control mice occupied the same UMAP space, indicating that monoallelic inactivation of *Vhl* had not affected their global transcriptional profiles. Interestingly, although distal tubular cells were present in tdTomato-negative samples, few were present in tdTomato-positive samples, suggesting that *Pax8-CreERT2* does not target this cell type at the given tamoxifen dose.

### A novel dichotomy in patterns of proximal tubular gene expression

Renal PT cells can be subdivided into S1 and S2 cells of the cortical convoluted tubule, and S3 cells of the outer medullary straight tubule (33). Unexpectedly, we found that each of these three cell types mapped to two distinct regions on the UMAP plot irrespective of tdTomato expression (Fig. 2A). These suggested that a determinant independent of established cell-type identities dichotomized gene expression in PT cells. To identify this determinant, we analyzed the 1,200 most variable genes within PT cells for mutually anticorrelated expression. Spearman correlations were calculated for every pair-wise combination of the variable genes and plotted onto a gene-by-gene matrix ordered by hierarchical clustering (Supplementary Fig. S1B). This revealed two areas with a high degree of anticorrelation, resulting from genes in clusters A to F (termed PT Module A) and clusters G and H (termed PT Module B; Supplementary Figs. S1C and S1D). Genes within each module are listed in Supplementary Table S1 with their respective correlation coefficients. Scoring cells for expression of these modules effectively segregated PT S1, S2, and S3 cells, but not non-PT cells, into two populations (Fig. 2C; Supplementary Fig. S1E). Spatially distinct expression of exemplar genes *Neat1* (for PT Module A) and *Fxyd2* (for PT Module B) demonstrated by dual RNA *in situ* hybridization on kidneys of Control mice confirmed that this cellular segregation was not an artefact of the scRNA-seq protocol (Fig. 2D). Cells with distinct *Neat1* or *Fxyd2* expression were often neighbors within the same tubule. We subsequently refer to cells with high PT Module A expression as “PT Class A” cells, and the others as “PT Class B” cells (Fig. 2E).

### Biallelic *Vhl* inactivation entrains early cell-specific transcriptomic changes in renal tubular cells

**Figure 3.**
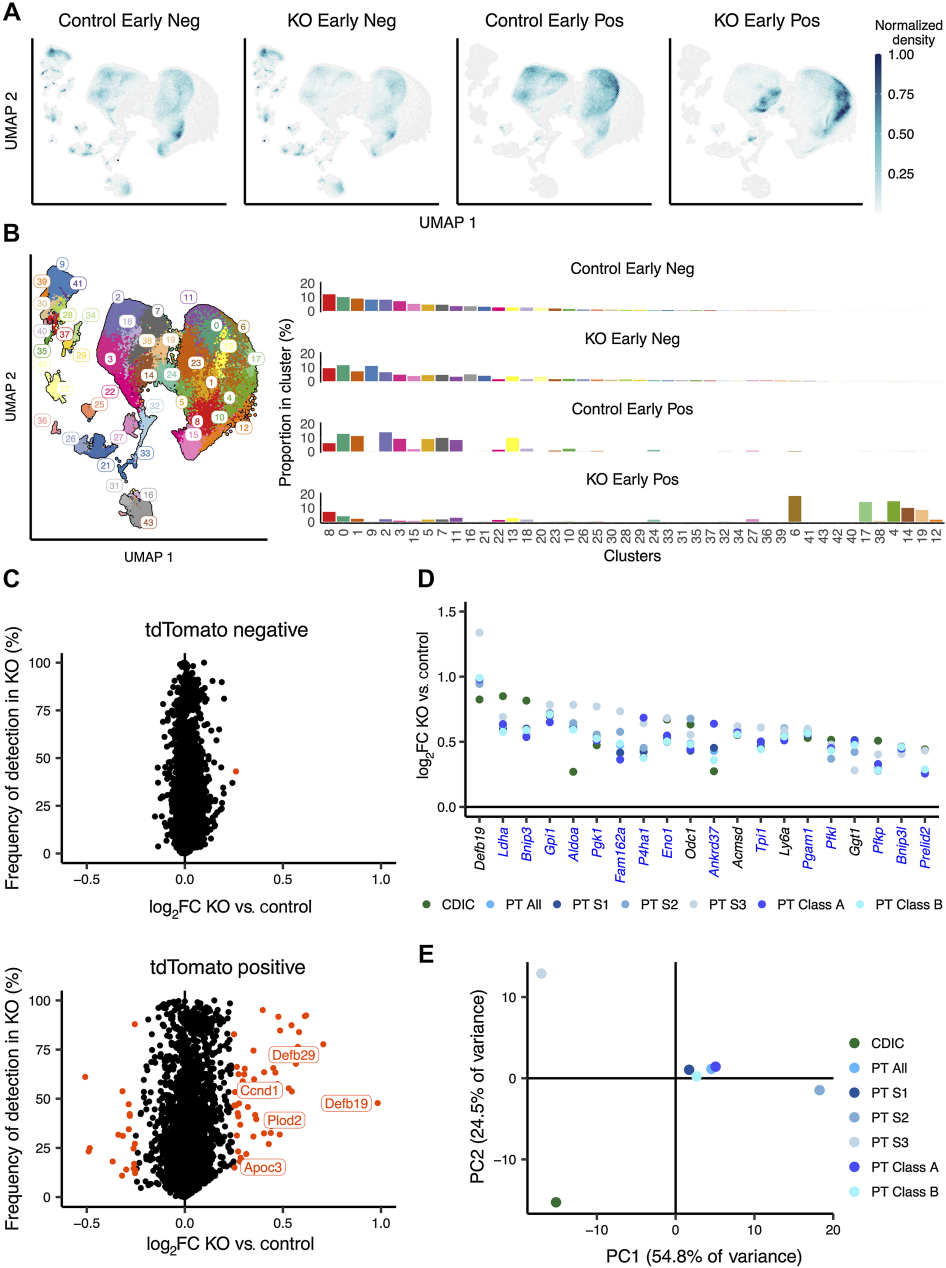
Biallelic *Vhl* inactivation entrains early cell-specific transcriptomic changes in RTE cells. **A,** Density plot depicting UMAP distribution of tdTomato-negative and -positive cells from kidneys of Control and KO mice harvested early after recombination. **B,** Left, UMAP plot depicting cells from Control and KO mice harvested early after recombination colored by UMAP clusters. Right, proportion of cells from each condition belonging to any cluster. **C,** Scatter plot depicting frequency of expression in tdTomato-negative (top) or tdTomato-positive (bottom) cells from KO mice against log_2_-fold change (log_2_FC) between cells from KO versus Control mice for all genes at the early time point. Orange, significantly regulated genes. Genes explicitly mentioned in the main text are labeled. **D,** Scatter plot depicting log_2_-fold change between tdTomato-positive cells from KO versus Control for genes significantly regulated in every renal cell identity. Blue, names of HIF target genes. **E,** PCA of gene expression changes early after *Vhl* inactivation in different renal cell identities. **A–E,** scRNA-seq data are shown for *n* = 3F, 1M for Control negative; *n* = 3F, 1M mice for Control positive samples; *n* = 2F, 1M mice for KO negative samples; *n* = 2F, 2M mice for KO-positive samples.

To assess if biallelic *Vhl* inactivation caused a shift in global transcriptomic profiles of cells, we compared the UMAP distribution of tdTomato-positive and -negative cells from KO or Control mice (Fig. 3A and B). tdTomato-negative cells from Control [*n* = 3 female (F), 1 male (M)] and KO (*n* = 2F, 1M) mice, essentially *Vhl^wt/wt^* and *Vhl^KO/wt^* respectively, occupied the same UMAP space. In contrast, tdTomato-positive cells from KO mice (*n* = 2F, 2M; essentially *Vhl^KO/KO^*) consistently occupied distinct positions on the UMAP and formed new clusters when compared with tdTomato-positive cells from Control mice (*n* = 3F, 1M; essentially *Vhl^wt/KO^*), indicating a specific transcriptomic change dependent on *Vhl* status.

To identify the genes driving this change, DE was tested between cells from KO and Control mice, which were either tdTomato-positive or tdTomato-negative. Testing was performed on all cells together, or on individual cell types separately. When all cells were analyzed together, 51 genes were upregulated in tdTomato-positive cells from KO compared with those from Control mice and 20 were downregulated (|log_2_ fold change| > 0.25, *P* < 0.01 after multiple testing correction; Fig. 3C; Supplementary Table S1). None of these genes were significantly altered in tdTomato-negative cells from KO compared with those from Control mice. Alterations in expression of these genes were highly correlated between individual KO samples, confirming their reproducibility (Supplementary Fig. S2A; Supplementary Table S1). Interestingly, KO mice of the same sex showed slightly higher correlation with one another than with the opposite sex (Supplementary Fig. S2A).

Next, we wanted to define the elements of these transcriptional changes that were common or specific to particular renal cell identities. Twenty genes were upregulated after *Vhl* inactivation in every cell identity analyzed, forming a “core” early response to *Vhl* inactivation in the RTE (Fig. 3D); none were commonly downregulated. 15 of these 20 genes had been previously characterized as HIF targets (see Materials and Methods; ref. 37), indicating that most of the core early response to *Vhl* inactivation is directly driven by HIF stabilization.

Interestingly, Defensin beta 19 (*Defb19*), not a known HIF target gene, was the most upregulated gene after *Vhl* inactivation in all PT cells and showed the second highest enrichment after lactate dehydrogenase A (*Ldha*) in CDICs. *Defb19* transcript could be detected readily in kidney tubules of KO mice but not Control mice with RNA *in situ* hybridization (Supplementary Fig. S2B), confirming upregulation. Notably, another member of the β-Defensin family, *Defb29*, was upregulated in every cell identity except PT S3, in which it narrowly missed the regulation threshold (log_2_-fold change = 0.23 instead of >0.25; Supplementary Table S1).

We then investigated the genes, other than those in the “core” response, that change following *Vhl* inactivation. A total of 177 genes were upregulated and 105 downregulated following *Vhl* inactivation in at least one cell identity (Supplementary Table S1). PCA segregated both PT S3 and CDIC cells from other cell identities based on changes in expression of these genes (Fig. 3E), indicating that these cell types exhibit distinct responses to *Vhl* inactivation. Only 1 of 29 and 2 of 14 genes that are specifically regulated in CDIC and PT S3 cells, respectively (Supplementary Figs. S2C and S2D) were previously characterized HIF targets (37). Notably, *Cdkn1a*, which codes for the cell-cycle inhibitory protein p21, and *Fbp1*, which codes for fructose bisphosphatase-1 identified as a tumor suppressor in ccRCC (41), were specifically upregulated following *Vhl* inactivation in CDIC, but not PT cells (Supplementary Fig. S2C).

PT Class A and PT Class B cells responded to *Vhl* inactivation in a similar manner, as exhibited by their proximity in the PCA plot (Fig. 3E). Consistent with this, all genes regulated by *Vhl* in PT Class B cells were also found to be regulated in PT Class A cells. However, 13 genes exhibited *Vhl*-dependent regulation in PT Class A but not PT Class B cells (Supplementary Fig. S2E), suggesting that PT Class A cells are more responsive to *Vhl* inactivation than PT Class B cells. Interestingly, of the 5 genes upregulated specifically in PT Class A cells that have known human orthologs, four are prognostic markers for ccRCC (unfavorable: *Adm, Vegfa, Ddit4*; favorable: *Igsf3*; Supplementary Fig. S2F). Collectively, these results demonstrate that early and direct consequences of *in vivo Vhl* inactivation are surprisingly specific to renal cell identity.

### Renal cortex, but not the papilla, is permissive for long-term survival of *Vhl*-null cells

To test whether these early consequences impacted the behavior and long-term survival of *Vhl-*null cells in the kidney, we compared the number and distribution of tdTomato-positive cells in four regions: cortex, outer medulla, inner medulla, and papilla—from Control and KO mice harvested at early and late time points.

**Figure 4.**
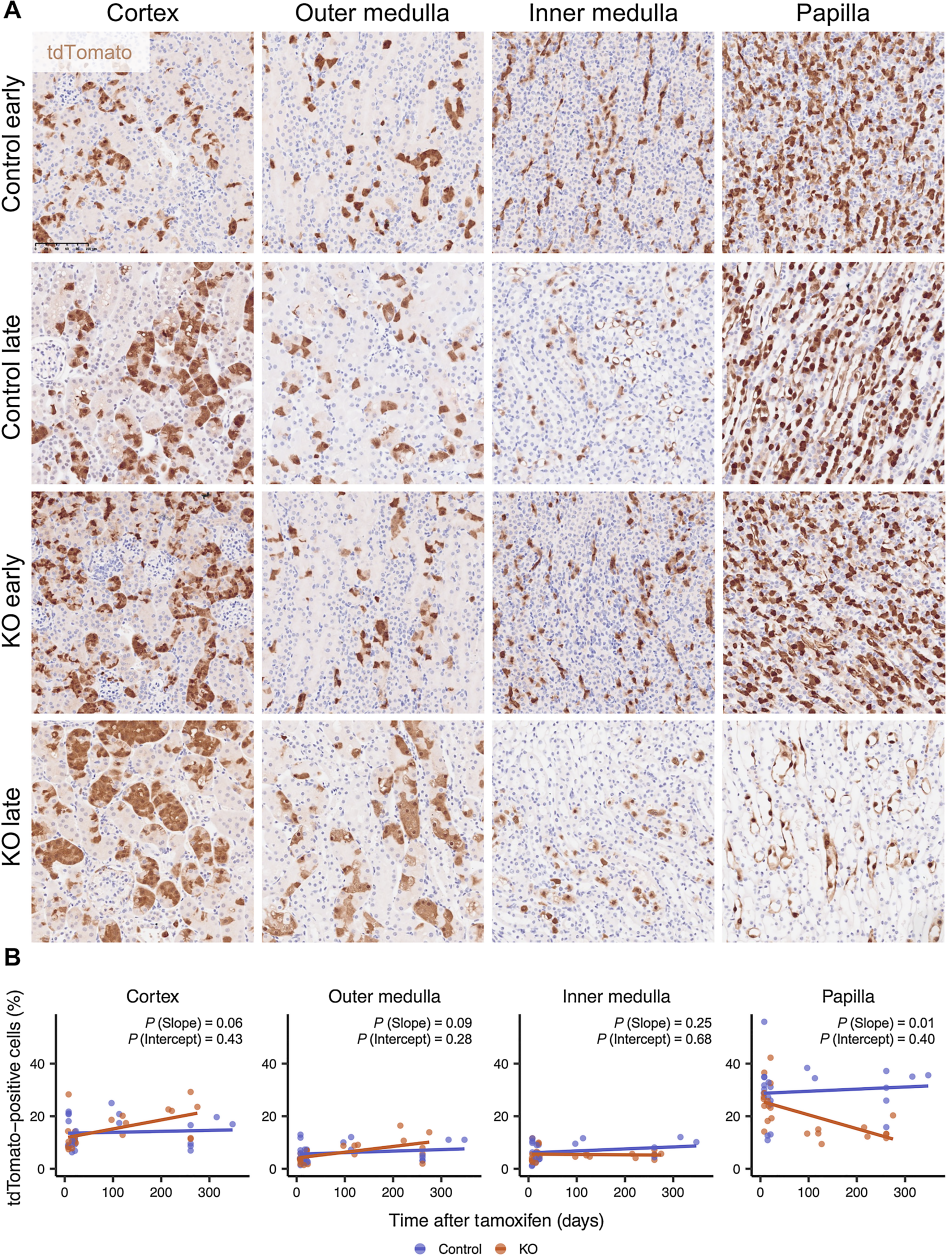
Renal cortex, but not the papilla, is permissive to long-term survival of *Vhl*-null cells. **A,** Representative tdTomato IHC counterstained with hematoxylin in different renal anatomical regions of Control or KO mice harvested at the early or late time points. Scale bar, 100 μm. Magnification, ×20. **B,** Proportion of cells that are tdTomato-positive in different regions of the kidney as quantified by tdTomato IHC in kidneys from Control or KO mice harvested at different intervals after recombination. *n* = 7F, 16M for all regions for KO; *n* = 9F, 15M; 9F, 15M; 9F, 14M; 8F, 14M for cortex, outer medulla, inner medulla, and papilla, respectively, for Control. Line denotes linear regression. Significance testing performed for slope and intercept of linear regression by *t* test.

tdTomato-positive cells were first compared in all regions of the kidneys in Control and KO mice at the early time point (Fig. 4A). No significant difference (*P* > 0.05) was detected in numbers of tdTomato-positive cells, indicating that recombination is not affected by the status (WT or KO) of the second *Vhl* allele (Fig. 4B). In kidneys of Control mice, the proportion of cells in each region positive for tdTomato remained the same, indicating that monoallelic *Vhl* inactivation conferred neither advantage nor disadvantage to cells in the RTE. In kidneys of KO mice, this proportion decreased significantly over time (*P* = 0.01) in the papilla. In contrast, the proportion exhibited an upward trend in the cortex (*P* = 0.06) and outer medulla (*P* = 0.09). Consistent with this increase, we observed that tdTomato-positive cells in the cortex and outer medulla of kidneys from KO mice often appeared as small clusters occupying most of the cells in a transverse section of many renal tubules at the late time point compared with being solitary at the early time point (Fig. 4A). However, a comparison of kidneys from KO and Control mice using hematoxylin and eosin, Trichrome, PAS, and ORO staining did not reveal clear abnormalities and the cells remained confined within the apparently normal renal tubule. We observed no dysplastic lesions or tumors within kidneys from KO mice at any time point. Collectively, these results indicate that biallelic *Vhl* inactivation confers a survival disadvantage to RTE cells in the renal papilla, but an advantage to those in the cortex and outer medulla that is still insufficient for tumor formation at least over the time course of this experiment.

### *Vhl*-null cells specifically undergo time-dependent alterations in gene expression

To identify gene expression changes associated with long-term survival of *Vhl*-null cells in tissue, we performed scRNA-seq on flow-sorted tdTomato-positive and -negative cells from KO and Control mice harvested at the late time point. This yielded data from an additional 242,561 high-quality cells across 16 samples from 9 mice, with an average of 15,160 ± 5367 (SD) cells, 3,232 ± 665 (SD) transcripts per cell that covered 1,254 ± 191 (SD) genes per cell per sample, which were integrated with data from cells from the early time point for subsequent analyses.

**Figure 5.**
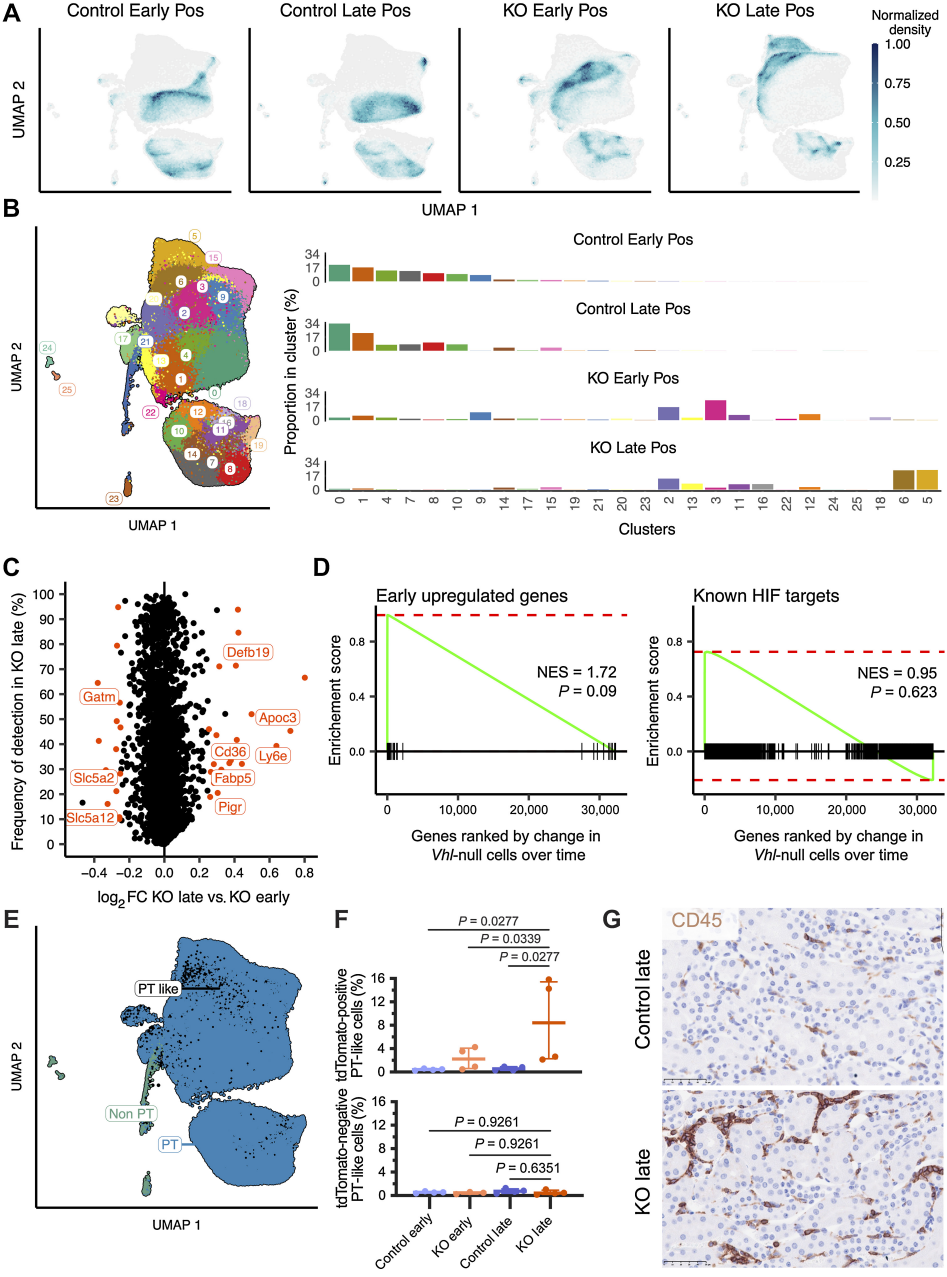
*Vhl*-null cells specifically undergo time-dependent alterations in gene expression. **A,** Density plot depicting UMAP distribution of tdTomato-positive cells from kidneys of Control and KO mice harvested at the early or late time points. **B,** Left, UMAP plot depicting tdTomato-positive cells from Control and KO mice harvested at the early and late time points colored by UMAP cluster. Right, proportion of cells from each condition belonging to any cluster. **C,** Scatter plot depicting frequency of expression in tdTomato-positive cells from KO mice at the late time point against log_2_-fold change (log_2_FC) between cells from KO mice at the late versus early time points for all genes. Orange, significantly regulated genes. Genes explicitly mentioned in the main text are labeled. **D,** Gene set enrichment plots depicting upregulation of genes regulated early after *Vhl* inactivation (left) or genes known to be HIF targets (right) in *Vhl*-null cells at the late versus early time points. NES, normalized enrichment score. *P* value adjusted by Bonferroni correction for multiple testing. **E,** UMAP plot depicting “PT like” cells among tdTomato-positive cells from Control and KO mice harvested at the early and late time points. Black, PT-like cells. **F,** Proportion of cells inferred to be “PT like” within tdTomato-positive (top) or tdTomato-negative (bottom) cells across conditions. Median and interquartile range plotted. Pairwise comparisons tested by one-way ANOVA with Holm–Šídák correction. **G,** Representative CD45 IHC on kidneys from Control (*n* = 1F, 4M) and KO (*n* = 6M) mice harvested at the late time point. Scale bar, 50 μm. Magnification, ×40. **A–F**, scRNA-seq data shown for *n* = 3F, 1M mice for Control early and Control late samples; *n* = 2F, 2M mice for KO early and KO late samples.

We compared gene expression by UMAP and performed DE analysis at late versus early time points in tdTomato-positive cells from KO mice and compared effects with those in Control mice. Different UMAP positions and cluster distributions were observed at the late versus early time point for cells from KO, but not from Control mice (Fig. 5A and B), indicating that *Vhl*-null cells undergo specific time-dependent changes in gene expression rather than these changes simply reflecting cell gains and losses. The differentially-expressed genes include 24 upregulated and 16 downregulated genes (Fig. 5C; Supplementary Figs. S3A and Supplementary Table S1; see Materials and Methods); *in situ* RNA hybridization is shown for *Apoc3* and *Defb19* in Supplementary Fig. S3B. Interestingly, the genes regulated over time were not significantly enriched for those regulated early after *Vhl* inactivation or those known to be HIF targets (Fig. 5D; Supplementary Fig. S3C).

Several genes considered to be markers of proximal tubule differentiation (*Slc5a2, Slc5a12, Gatm*) were downregulated in *Vhl*-null cells over time, suggesting a loss of PT identity. To test this, we examined the data for cells that clustered with PT cells but were not assigned as a PT cell type (see Materials and Methods). Our data contained 7,673 such cells that we called “PT like” (Fig. 5E). *Vhl*-null cells at the late time point contained a significantly higher (*P* = 0.03) proportion of PT like cells compared with other conditions (Fig. 5F). Compared with PT cells, PT like cells showed significant (*P* = 4.6×10^−8^) downregulation of PT marker genes (Supplementary Fig. S3D; Supplementary Table S1), but no upregulation of marker genes of any non PT cell type. Notably, the PT developmental transcription factor (42) *Maf* was downregulated (*P* < 1 × 10^−16^) in these cells but the pan-RTE transcription factor (43) *Pax8* remained unchanged (*P* > 0.01; Supplementary Fig. S3E). PT like cells contained a significantly higher proportion of cycling cells than other PT identities, as measured by the number of cells expressing the proliferation marker *Mki67* (Supplementary Fig. S3F; ref. 44). Taken together, these results indicate that *Vhl* inactivation causes PT cells to de-differentiate over time.

Genes upregulated over time included *Apoc3*, *Cd36*, and *Fabp5*, all of which are involved in lipid and fatty acid metabolism. They also included *Ly6e*, *Pigr*, and particularly *Defb19*, which was already upregulated early after *Vhl* inactivation. These three genes code for surface or secreted proteins predicted to communicate with immune cells (45–47). In line with this, we observed leukocyte infiltration in kidneys from KO mice at the late time point as evidenced by IHC with the pan-leukocyte marker CD45 (Fig. 5G; Supplementary Fig. S3G). Associated with this, we identified time-dependent gene expression changes in tdTomato-negative immune cell populations within kidneys from KO mice, with macrophages in particular upregulating genes involved with leukocyte activation (*P* = 0.0068; Supplementary Fig. S3H).

Taken together, these results demonstrate that biallelic inactivation of *Vhl* leads to changes in gene expression in affected cells, which evolve after the initial HIF-dependent response. These changes appear sufficient to drive immune and inflammatory changes in the renal interstitium, at least in this setting.

### *Vhl*-null cells exhibit time-dependent proliferation

**Figure 6.**
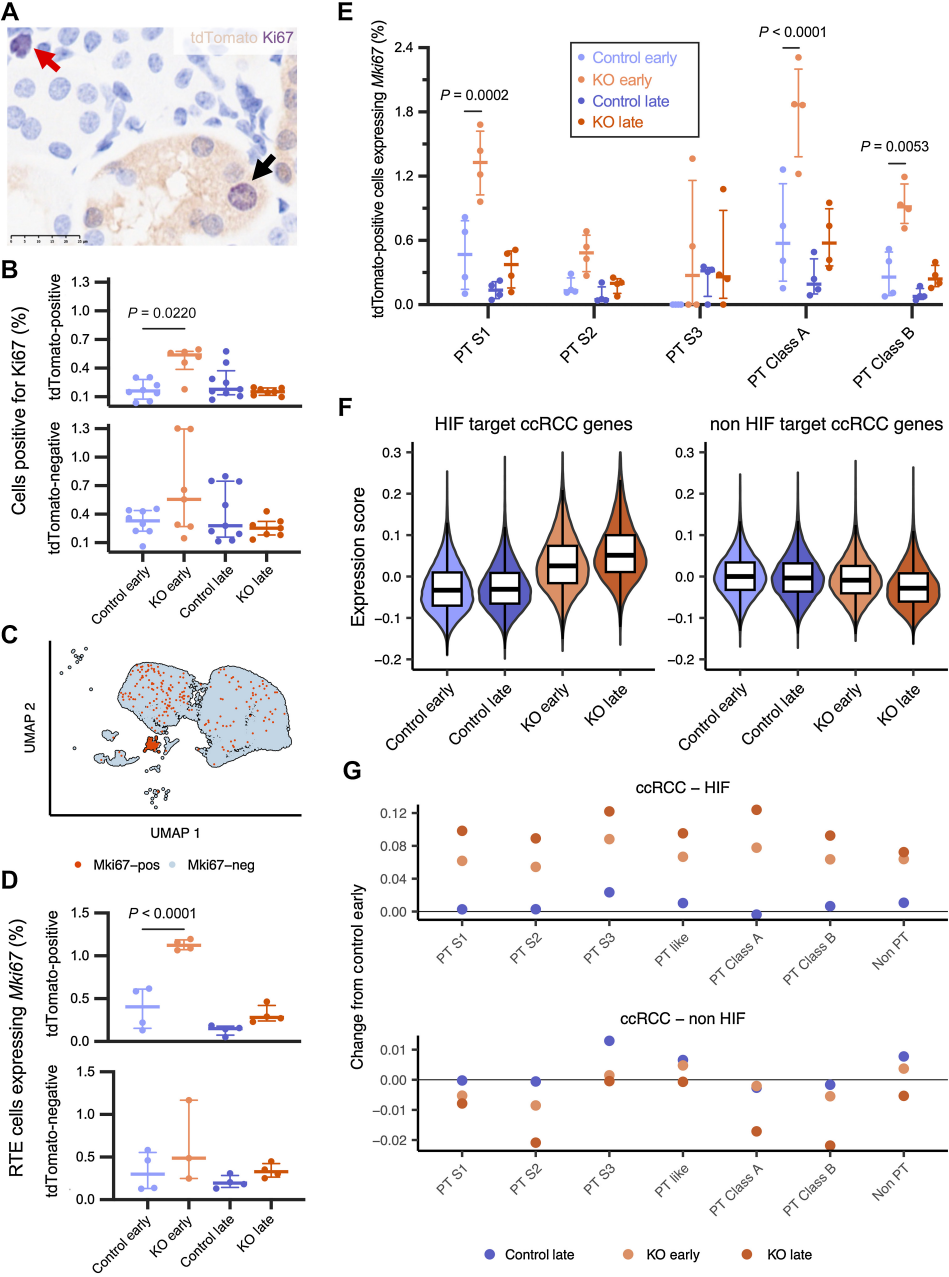
*Vhl*-null cells exhibit time-dependent proliferation and association with ccRCC-like gene expression. **A,** Representative dual IHC for tdTomato (brown) and Ki67 (purple) counterstained with hematoxylin in kidneys of KO mice harvested early after recombination. Scale bar, 25 μm. Magnification, ×40. Black arrow, dual-positive cell; red arrow, tdTomato-negative Ki67-positive cell. **B,** Proportion of tdTomato-positive (top) or tdTomato-negative (bottom) cells that are positive for Ki67 by dual IHC in kidneys of Control and KO mice harvested early and late after recombination (*n* = 2F, 6M for Control early; *n* = 4F, 2M for KO early; *n* = 4F, 5M for Control late; *n* = 1F, 6M for KO late). Pairwise comparisons by Kruskal–Wallis test with Dunn correction. **C,** UMAP plot depicting RTE cells from Control and KO mice at the early and late time points. Orange, cells expressing *Mki67*. **D,** Proportion of tdTomato-positive (top) or tdTomato-negative (bottom) RTE cells that express *Mki67* in different conditions. Pairwise comparisons tested by one-way ANOVA with Holm–Šídák correction. **E,** Proportion of tdTomato-positive cells of different PT identities that express *Mki67* in different conditions. Pairwise comparisons tested by two-way ANOVA with Holm–Šídák correction. **F,** Violin plot overlaid with boxplot depicting expression score for genes upregulated in ccRCC cells known to be HIF targets (left) and not known to be HIF targets (right) in tdTomato-positive cells from Control and KO mice harvested at early or late time points. **G,** Scatter plot depicting changes in mean expression scores for HIF-target (top) and non-HIF-target (bottom) genes specifically upregulated in ccRCC, in tdTomato-positive cells of different PT identities from different conditions when compared with those from Control mice at the early time point. **B, D,** and **E**, Median and interquartile range plotted. Only significant (*P* < 0.05) comparisons shown. **C–G,** scRNA-seq data shown for *n* = 3F, 1M mice for tdTomato-positive and tdTomato-negative Control early and Control late samples; *n* = 2F, 1M mice for tdTomato-negative KO early samples; *n* = 2F, 2M mice for tdTomato-positive KO early samples; *n* = 2F, 2M mice for tdTomato-positive and tdTomato-negative KO late samples.

The apparent selective advantage conferred by *Vhl* inactivation may reflect an increase in the proliferative capacity of *Vhl*-null cells. To test this, we performed dual IHC for tdTomato and the cell proliferation marker Ki67 (48) in kidneys from Control and KO mice harvested at early or late time points. The proportion of tdTomato-positive or -negative cells in the renal cortex that were positive for Ki67 was quantified. A significantly greater (*P* = 0.02) proportion of tdTomato-positive cells were Ki67-positive in the cortex of KO compared Control mice at the early (Fig. 6A and B; see Materials and Methods), but not at the late time point (*P* > 0.99). In contrast, the proportion of tdTomato-negative cells positive for Ki67 in kidneys of KO mice was not significantly different to those of Control mice at either early (*P* = 0.73) or late (*P* > 0.99) time points. Together, these results indicate that although *Vhl* inactivation in the RTE is associated with a specific entry of *Vhl*-null cortical cells into cell cycle early on, it is not sustained over time.

The transient nature of this proliferative burst could also be inferred from analysis of RTE cells identified in the scRNA-seq data. Consistent with the IHC data, a greater (*P* < 0.0001) proportion of tdTomato-positive RTE cells were in cell cycle in KO mice compared with RTE cells in Control mice at the early time point as assessed by their transcriptional profile (Fig. 6C and D); no such increase was evident at the late time point. Upregulation of Cyclin D1 (*Ccnd1*) was also observed in tdTomato-positive cells from KO compared with Control mice at the early time point (Fig. 3C). The transient proliferative phenotype was observed in all PT cell identities except for PT S2 and S3; interestingly, the early proliferative burst was most significant (*P* < 0.0001) in PT Class A cells (Fig. 6E).

### Associations with ccRCC-like transcriptomic profiles

Finally, to determine if there were overlaps between the changes in *Vhl*-null cells and a “ccRCC-like transcriptomic profile,” a list of genes specifically upregulated in tumor cells compared with normal proximal tubular cells was generated by reference to published scRNA-seq studies on ccRCC samples (see Materials and Methods; ref. 38). Genes that were upregulated in ccRCC were categorized on the basis of their known regulation by HIF (37), and their expression was scored in tdTomato-positive cells from KO and Control mice at early and late time points. Expression of ccRCC-upregulated HIF target genes was higher (*P* < 1×10^−16^) in tdTomato-positive cells from KO than from Control mice at the early time point, with a further modest increase over time (Fig. 6F). All PT and PT like cells exhibited this increase, with PT Class A cells exhibiting the highest response (Fig. 6G). Interestingly, expression of ccRCC-upregulated genes not known to be HIF targets remained unchanged early after *Vhl* inactivation but decreased significantly (*P* < 1 × 10^−16^) over time (Fig. 6F and G). Thus, although *Vhl*-null cells immediately upregulate ccRCC-associated HIF-target genes, they downregulate other ccRCC-associated genes over time.

## Discussion

Here we present a new experimental strategy for understanding oncogenic pathways, in which cells bearing putative oncogenic mutations are generated in their native context, marked, and analyzed at cellular resolution over time. Focusing on the *VHL* tumor suppressor gene (13), we designed a mouse allele in which conditional inactivation of *Vhl* is inherently linked to the activation of a tdTomato fluorescent reporter. Direct coupling of *Vhl* inactivation and reporter activation avoids the differential action of Cre recombinase on separate alleles that limits accuracy in binary Cre-recombinase/lox-STOP-lox reporter lines (49). Our model does not intrinsically allow individual cells to be followed through clonal development. Nor can this be readily inferred from spatial relationships of cells studied at the different time points. However, it provides an accurate platform for the *in vivo* analysis and *ex vivo* retrieval of marked cells at different intervals following *Vhl* inactivation.

Both sporadic and inherited ccRCC are associated with biallelic inactivation of *VHL* (9). In VHL disease, one defective *VHL* allele is inherited, and the other is lost somatically (50). We modeled this by generating mice with one conditional reporter allele and one constitutively inactivated allele of *Vhl* (*Vhl*^*jae.KO/pjr.fl*^; ref. 25). In mice that also expressed the inducible Cre recombinase transgene, *Pax8*-*CreERT2*, timed activation of Cre converted monoallelic *Vhl* inactivation to biallelic inactivation. Mice bearing this conditionally inactivated *Vhl* allele, but in association with a wild-type *Vhl* allele (*Vhl^wt/pjr.fl^*) provided a comparator against which the evolution of changes following biallelic *Vhl* inactivation could be assessed. Using this system, we describe for the first time the effects of *Vhl* inactivation at cellular resolution in the native kidney context. Our findings add to the understanding of *Vhl*-associated tumor suppressor functions in several different ways.

They suggest that the tissue specificity of *Vhl*-associated neoplasia may have its origins in very early events after *Vhl* inactivation and potentially provide new insights into the cell of origin. Single cell transcriptomic analyses prior to *Vhl* inactivation agreed well with those previously reported for mouse kidney (20–23). However, using externally derived markers to identify different nephron segments independently of grouping by dimension reduction, our analyses also revealed an unexpected dichotomy in gene expression within renal proximal tubular cells, which cut across patterns of gene expression identified in PT (S1, S2 and S3 segments) in this, and other single-cell studies (20–23). This clearly identified two populations, distinguished in part by expression of long noncoding RNAs *Neat1* and *Malat1*. The presence of these two populations was supported by *in situ* verification of dichotomous *Neat1* expression in renal tubules. We cannot distinguish whether differences represent distinct differentiation pathways or interchangeable cell states. Nevertheless, these populations, which we have termed Class A and B PT cells, behaved differently in respect of *Vhl* inactivation, in a manner which suggested that Class A cells may be the source of neoplasia. PT Class A cells exhibited a greater magnitude of changes in *Vhl*-dependent genes, specific regulation of genes associated with adverse prognosis of ccRCC, and manifested the greatest increase in expression of ccRCC-associated genes that are known transcriptional targets of HIF. Furthermore, these cells were associated with a greater prevalence of markers of cycling cells. Previous studies have associated ccRCC cells of origin with PT cells expressing the cell adhesion molecule *VCAM1* (19, 51). While *Vcam1* was not identified in our analysis, we observed overlap in genes that define Class A PT cells and those that were differentially enriched in *VCAM1*-positive human PT cells.

Aside from these findings, single-cell transcriptomic analyses of cells across different renal epithelial cell identities revealed a surprising level of heterogeneity in response to *Vhl* inactivation. The *Vhl* ‘marking’ strategy also enabled us to define changes in the numbers of *Vhl*-null cells, irrespective of any morphological abnormality. These analyses revealed striking changes over time in the numbers of surviving *Vhl*-null cells in different regions of the kidney. In the cortex and outer medulla, principally composed of proximal tubules (52), the number of *Vhl*-null cells increased substantially. In contrast, a marked decrease in *Vhl*-null cells was observed in the papilla. This indicated that some renal epithelial populations may be intolerant of *Vhl* inactivation even in their native context, again potentially contributing to the tissue specificity of *VHL*-associated oncogenesis.

Based on the absence of neoplastic lesions in unmarked models of Vhl inactivation (53–55), it has been assumed that *Vhl* inactivation has no oncogenic action without inactivating mutations in one or more other genes such as *Pbrm1* or *Bap1* (28, 56–58). These genes reside on human chromosome 3p and are commonly inactivated as part of the initiating chromothripsis event in human RCC and/or subsequently mutated (9, 59). Nonetheless, the truncal nature of *VHL* mutation in ccRCC evolution, and the long latency postulated between biallelic *VHL* inactivation and *PBRM1*/*BAP1* inactivation (9, 59) suggest that time-accrued effects of *VHL* inactivation inform the oncogenic trajectory of at least some renal cells. With the caveat that we cannot identify individual cells that are on a certain trajectory to cancer, detailed analysis of marked *Vhl*-null cells revealed multiple effects of potential relevance to *VHL*-associated oncogenesis.

Our analyses of *Vhl* inactivation were focused on two time points: early (1–3 weeks) and late (4–12 months) that were respectively designed to capture early effects of *Vhl* inactivation such as activation of the HIF transcriptional cascade or track delayed effects. HIF is rapidly activated in hours following loss of VHL-dependent proteolysis and a fully activated HIF transcriptional cascade might be expected in days (60). Unexpectedly however, although no further interventions were performed, *Vhl*-null cells manifested marked late changes in gene expression as revealed by analyses at the later time points. These changes did not reflect an obvious gain or loss of amplitude of the HIF transcriptional response but rather reflected two principal changes: reduced expression of proximal tubular differentiation genes and induction of genes involved in lipid metabolism, both of which are observed in *VHL*-associated ccRCC.

Interestingly, one of the most marked changes in gene expression at both early and late time points was in transcripts encoding β-Defensins. *Vhl* inactivation was associated with early upregulation of Defensin beta 19 (*Defb19*) and 29 (*Defb29*) and a further increase in *Defb19* expression in *Vhl*-null cells was observed over time. β-Defensins are secreted proteins that signal to the innate immune system (47). Associated with this, increased leukocyte infiltration and increased expression of genes associated with immune activation were observed in the renal interstitium. Remarkably, although early after *Vhl* inactivation there was almost no interstitial change, these immune and inflammatory changes were always observed at later time points, suggesting that they occur as a direct, albeit delayed, consequence of *Vhl* inactivation rather than requiring neo-antigen presentation following the accrual of additional mutations.

Of particular interest in relation to *Vhl*-associated oncogenesis was a time-limited proliferation observed in *Vhl*-null PT cells. At the early time point this was evidenced by IHC and single-cell transcriptomic markers of cycling cells and by increases over time in the number of marked *Vhl*-null PT cells in cortical and outer medullary regions. These findings support a cell type–specific and autonomous effect of *Vhl* inactivation on cell proliferation. However, to our surprise, markers of proliferation had subsided almost to baseline levels at the later time points, and we did not observe dysplastic or neoplastic renal tubules. We do not understand the mechanisms constraining this proliferation though transient cell proliferation driven by the activation of HIF has been described in other tissues including the carotid body (61) and lungs (62). Interestingly however, we did observe that, at later time points, “marked” *Vhl*-null cells within nephron sections appeared much more frequently to encompass the entire renal tubular section, but that these renal tubules remained morphologically normal. One possibility is that the constraints of tubular anatomy (63) are responsible for limiting proliferation. Another possibility is that β-Defensins and/or associated immune cell and inflammatory changes in the renal interstitium might generate antiproliferative signals.

Further work will now be required to understand the mechanisms underlying this early proliferation and its restraint. To conclude, this work describes a new approach to the definition of early oncogenic effects in intact tissues that couples precise cell marking to time-resolved analyses. We illustrate its ability to define hitherto unforeseen events following *Vhl* inactivation in the native renal epithelium, which open new insights into the actions of this enigmatic tumor suppressor.

## Supplementary Material

Supplementary Table S1 scRNA-seq metrics, cell type markers, and lists of differentially expressed genes

Supplementary Figure S1 Single-cell RNA sequencing on flow-sorted renal cells

Supplementary Figure S2 Biallelic Vhl loss entrains early cell-specific transcriptomic changes in renal tubular cells

Supplementary Figure S3 Vhl-null cells specifically undergo time-dependent alterations in gene expression
