# Supplementary material for: Oncogenic Cell Tagging and Single-Cell Transcriptomics Reveal Cell Type–Specific and Time-Resolved Responses to Vhl Inactivation in the Kidney
Source: Cancer Res. 2024 Mar 19;84(11):1799–816. doi: 10.1158/0008-5472.CAN-23-3248 (PMC11148546; doi:10.1158/0008-5472.CAN-23-3248)
Supplement: Supplementary Figure S1 — Single-cell RNA sequencing on flow-sorted renal cells [file can-23-3248_supplementary_figure_s1_suppsf1.pdf]

Supplementary Figure S1

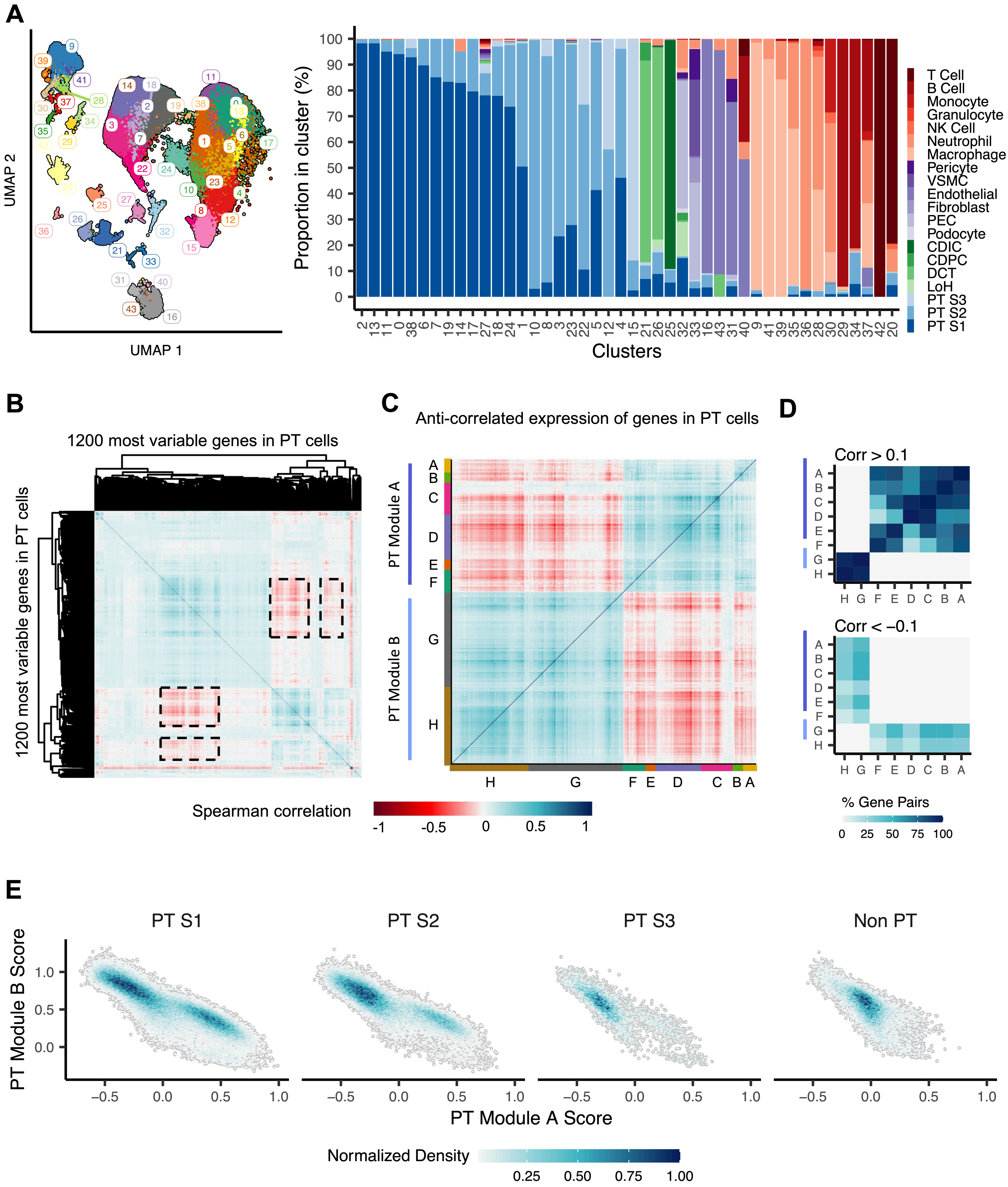

Supplementary Figure S1: Single-cell RNA sequencing on flow-sorted renal cells. **(A)** UMAP plot depicting cells from kidneys of Control mice colored by UMAP clusters (left). Cell-type composition of each cluster (right). PT – Proximal Tubule; LoH – Loop of Henle; DCT – Distal Convoluted Tubule; CD – Collecting duct, PC – Principal Cell, IC – Intercalated; PEC – Parietal epithelial cell; VSMC – Vascular Smooth Muscle Cell; NK – Natural Killer cell. **(B)** Correlation heatmap for 1,200 most-variable genes expressed in PT cells in kidneys of Control mice after hierarchical clustering. Two areas with high degree of anti-correlation marked by black rectangles. **(C)** Heatmap depicting pairwise Spearman correlations that were highlighted by black rectangles in (B). Hierarchical clusters of genes depicting anti-correlated expression are labelled A-F (PT Module A) and G-H (PT Module B). **(D)** Proportion of gene-pairs between each cluster of genes with Spearman correlations above 0.1 (top) or below -0.1 (bottom). **(E)** Density plot depicting distribution of PT S1, PT S2, PT S3, and non-proximal tubular (Non PT) cells based on the expression scores for PT Module B against PT Module A. **(A-E)** scRNA-seq data shown for n = 3 female (3F) and n = 1 male (1M) Control mice.
