# Supplementary material for: Oncogenic Cell Tagging and Single-Cell Transcriptomics Reveal Cell Type–Specific and Time-Resolved Responses to Vhl Inactivation in the Kidney
Source: Cancer Res. 2024 Mar 19;84(11):1799–816. doi: 10.1158/0008-5472.CAN-23-3248 (PMC11148546; doi:10.1158/0008-5472.CAN-23-3248)
Supplement: Supplementary Figure S2 — Biallelic Vhl loss entrains early cell-specific transcriptomic changes in renal tubular cells [file can-23-3248_supplementary_figure_s2_suppsf2.pdf]

Supplementary Figure S2

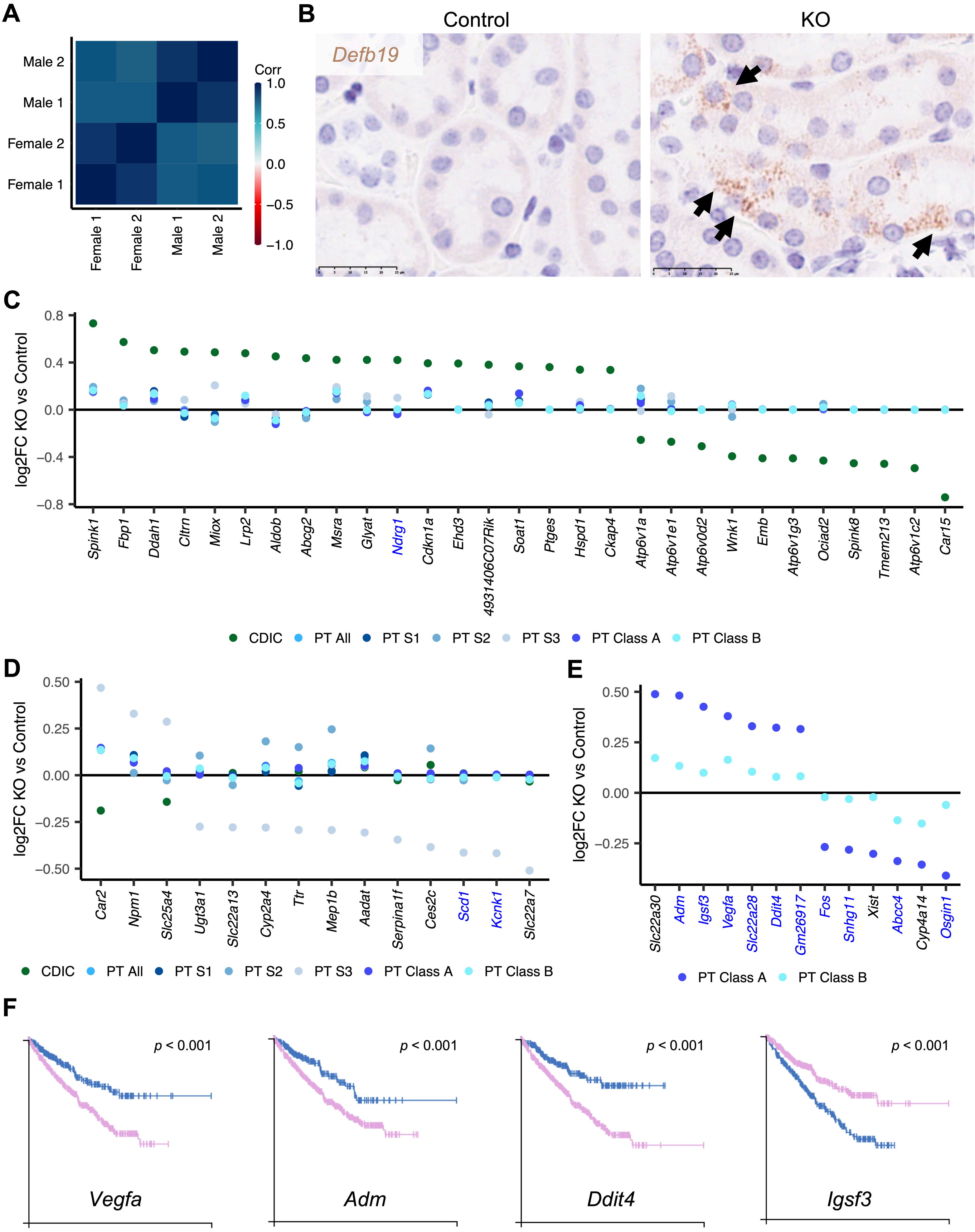

Supplementary Figure S2: Biallelic *Vhl* loss entrains early cell-specific transcriptomic changes in renal tubular cells. **(A)** Heatmap depicting Spearman correlation coefficients for changes in *Vhl*-regulated genes in tdTomato-positive cells derived from individual KO mice. **(B)** Representative *in situ* RNA hybridization for *Defb19* mRNA in kidneys from Control or KO mice (n = 2F, 2M per genotype) harvested at the early timepoint. *Defb19* signal depicted by black arrows. Scale bar denotes 25 µm; 40x magnification. **(C-E)** Scatter plot depicting log_2-fold change (log2FC) between tdTomato-positive cells from KO *versus* Control for genes specifically regulated in **(C)** CDIC, **(D)** PT S3 cells, and **(E)** PT Class A cells. Names of HIF target genes (blue). **(F)** Survival analysis of renal cancer patients stratified by expression of genes specifically regulated in PT Class A cells after *Vhl* inactivation. Cases with high (blue) and low (pink) expression. Data from The Human Protein Atlas, accessed on 23 July 2023. **(A-E)** scRNA-seq data shown for n = 3F, 1M mice for Control positive samples; n = 2F, 2M mice for KO positive samples.
