# Supplementary material for: Oncogenic Cell Tagging and Single-Cell Transcriptomics Reveal Cell Type–Specific and Time-Resolved Responses to Vhl Inactivation in the Kidney
Source: Cancer Res. 2024 Mar 19;84(11):1799–816. doi: 10.1158/0008-5472.CAN-23-3248 (PMC11148546; doi:10.1158/0008-5472.CAN-23-3248)
Supplement: Supplementary Figure S3 — Vhl-null cells specifically undergo time-dependent alterations in gene expression [file can-23-3248_supplementary_figure_s3_suppsf3.pdf]

# Supplementary Figure S3

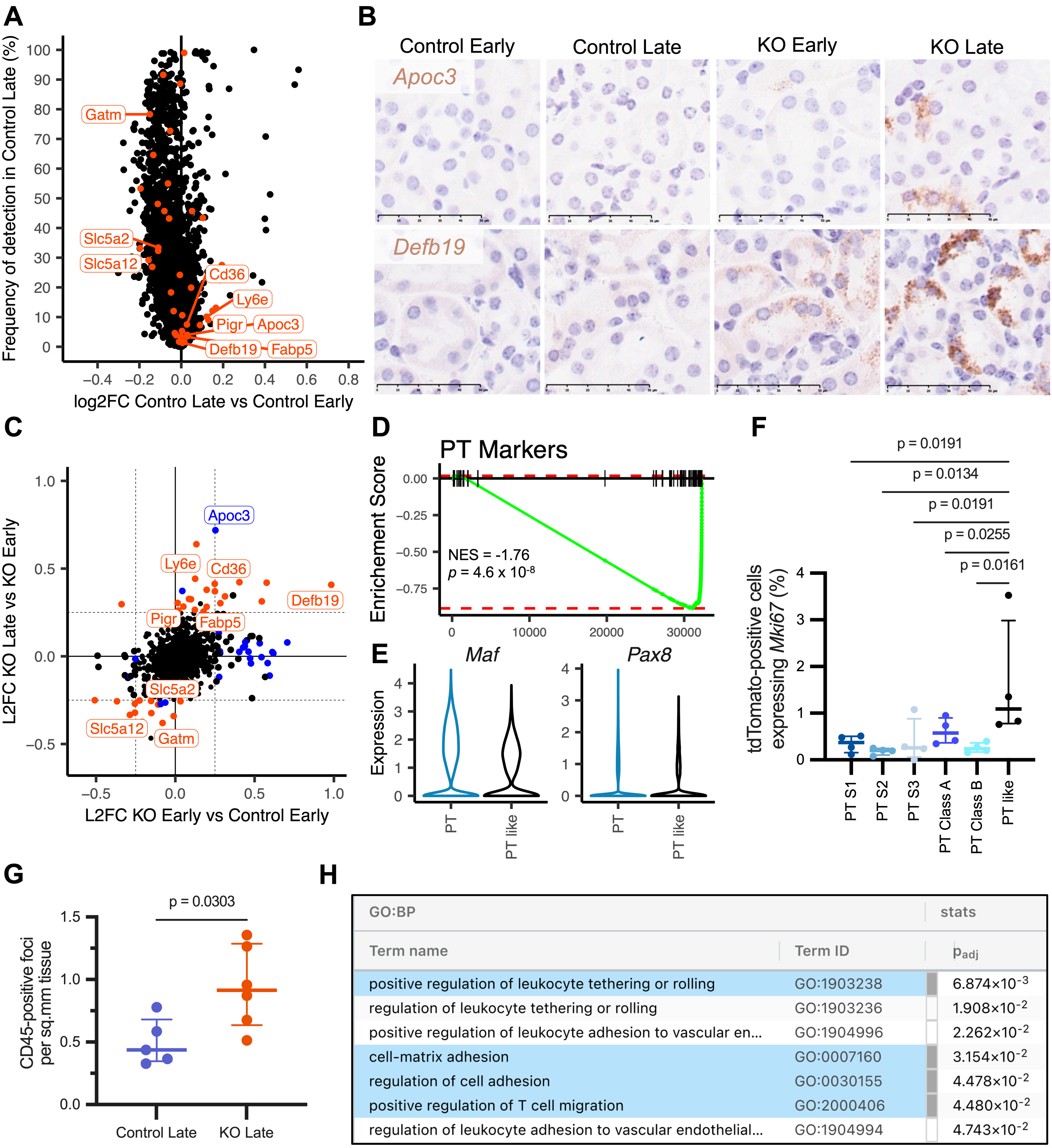


| GO:BP | | stats |
|---|---|---|
| Term name | Term ID | $p_{adj}$ |
| positive regulation of leukocyte tethering or rolling | GO:1903238 | $6.874\times10^{-3}$ |
| regulation of leukocyte tethering or rolling | GO:1903236 | $1.908\times10^{-2}$ |
| positive regulation of leukocyte adhesion to vascular en... | GO:1904996 | $2.262\times10^{-2}$ |
| cell-matrix adhesion | GO:0007160 | $3.154\times10^{-2}$ |
| regulation of cell adhesion | GO:0030155 | $4.478\times10^{-2}$ |
| positive regulation of T cell migration | GO:2000406 | $4.480\times10^{-2}$ |
| regulation of leukocyte adhesion to vascular endothelial... | GO:1904994 | $4.743\times10^{-2}$ |

**Supplementary Figure S3: *Vhl*-null cells specifically undergo time-dependent alterations in gene expression**. **(A)** Scatter plot depicting frequency of expression in tdTomato-positive cells from Control mice at the late timepoint against $\log_2$-fold change (log2FC) between cells from Control mice at the late *versus* early timepoints for all genes. Genes specifically regulated over time in *Vhl*-null cells (orange; from Fig 5C). Genes explicitly mentioned in the main text are labelled. **(B)** Representative *in situ* RNA hybridization for *Apoc3* (top) and *Defb19* (bottom) mRNA in kidneys from Control and KO mice harvested at the early and late timepoints (at least n = 2F, 1M per timepoint for both transcripts). Scale bar denotes 50 µm; 40x magnification. **(C)** Scatter plot depicting $\log_2$-fold change (L2FC) in *Vhl*-null cells over time against $\log_2$-fold change early after *Vhl* inactivation for all genes. Genes regulated over time (orange). HIF target genes (blue). Genes explicitly mentioned in the main text are labelled. **(D)** Gene set enrichment plot depicting downregulation of PT marker genes in PT-like cells compared to PT cells at the late timepoint. NES = Normalized Enrichment Score. p value adjusted by Bonferroni correction for multiple testing. **(E)** Violin plots depicting expression of *Maf* and *Pax8* in *Vhl*-null PT or PT like cells at the late timepoint. **(F)** Proportion of tdTomato-positive cells of any PT identity that express *Mki67* from kidneys of KO mice harvested at the late timepoint (n = 2F, 2M). Pairwise comparisons by one-way ANOVA with Holm-Šídák correction. **(G)** Number of foci of leukocytes observed per $mm^2$ of renal cortex and outer medulla in Control (n = 1F, 4M) and KO (n = 6M) mice harvested at the late timepoint. Pairwise comparison by Mann-Whitney test. **(H)** Significantly enriched Gene Ontology terms for genes upregulated over time in tdTomato-negative macrophages in KO samples. Results from gProfiler. **(F, G)** Median and inter-quartile range plotted.
